# Supplementary material for: Performance barriers of Civil Registration System in Bihar: An exploratory study
Source: PLoS One. 2022 Jun 1;17(6):e0268832. doi: 10.1371/journal.pone.0268832 (PMC9159592; doi:10.1371/journal.pone.0268832)
Supplement: S1 Text — (DOCX) [file pone.0268832.s002.docx]

**Guides for KII and FGD**

**Key informant interview - Medical Officers and Health Assistant**

1. Background characteristics

Age, sex, residence, education, type of health facilities, years of working experiences

1. What is the role of medical officer/health assistant in birth/death registration?
2. Who are the other stakeholders involved at PHCs in terms of birth and death registration?
3. How does birth and death certification perform within and outside hospitals?
4. Is there any law which regulate birth/death registration in this area? If yes, what are they?
5. Is there any document required for birth/death registration? If yes, what are those documents?
6. Is birth registration and issue of certificate done before discharge from PHCs or parents need to come again? What is the time gap between registration of birth/death and issue of certificate in your hospital?
7. Is there any gap between the number of deliveries reported and number of births registered at the health centre? If yes, what are the possible reason? How do you clear backlog, if any?
8. Is there any evaluation method for completeness of birth/death registration in this hospitals? If Yes, what is the method? How frequently do you evaluate?
9. How do you evaluate the quality of cause of death data reported in this area?
10. Is there any HR and logistics gap? If yes, what steps have been taken to overcome it?
11. What support do you receive from DSO/BSO in implementation of vital statistics system?
12. Is there any supervision? Any regular report review or meeting for smooth functioning of vital registration?
13. Have you and other health assistant received any training on registering vital events? If yes, when did you receive last training and what was the content?
14. What do you think about awareness, knowledge and attitude of the people towards registration of birth/death?
15. The success of registration of vital event to a great extend depends on awareness level of the communities? Were there any activities undertaken by you and other staffs of this hospital? If yes, please explain.
16. What are the challenges for you and other staffs faced in registration of birth/death and issue of certificate? Please explain in detail.
17. What are the suggestions for further improving the level of the birth/death in the PHCs?

**KII-BSO (Block Statistical Officer)**

1. Background characteristics

Age, Sex, education level, Position, Years of experience

1. What role do you have in overall management of vital registration system?
2. What is the target and coverage of your block?
3. What are the method of setting target and monitoring completeness of registration?
4. How do you get information on number of institutional deliveries? Is there any gap between the number of deliveries reported and number of births registered at the health institutions? If yes, what are the possible reasons? What actions are undertaken to reduce this gap?
5. How do you monitor birth registration in case of home deliveries?
6. How do you manage delay registration and, what step do you take to decrease such registration?
7. What are the necessary logistics required for registration of birth/death? Is there any gap in HR and logistic required for smooth functioning of this system?
8. What support do you received from district office for strengthened implementation of birth/death registration?
9. Is there any separate funding for smooth function of vital statistics system? Could you please explain different funding required for this system?
10. Have you received any training pertaining to birth and death registration? If yes, when did you receive last training and, what was the content of training?
11. Is there any time gap between birth/death registration and issue of birth certificate? If yes, what are the possible factors?
12. Is there any significant difference between block in terms of birth/death registration? If yes, what are the possible factors?
13. Do communities perceive any benefits of registering vital events? If yes, what are the perceived benefits?
14. What are the suggestions for further improving the level of birth/death registration in the districts?

**KII-Gram Panchayat/ Nagar Nigam**

1. Background characteristics

Age, Sex, education level, position and years of experience

1. What is the role of gram Panchayat/Nagar Nigam in terms of birth/death registration?
2. Who are the other stakeholders involved? What are their roles and responsibilities?
3. What are the documents required for registering vital events?
4. Is there any way to know the number of children not having birth registration? If yes, what is that way and how frequently the system updates? How do you address issue of delay registration?
5. What is the time gap between birth registration and issuance of birth certificate? What are the reason for the gap and what could be done to reduce this gap?
6. What are the necessary logistics required for registration of birth/death? Is there any gap in HR and logistic required for smooth functioning of this system?
7. What support do you receive from block or district office for smooth functioning of birth/death registration?
8. Have you received any training on birth/death registration? If yes, when did you receive last training and, what was the content of such training?
9. Are review meetings on birth and death registration held at block or district level? If yes, what are frequency of such meetings/ what issues are generally discussed? Is there any feedback mechanism?
10. Do communities perceive any benefits of registering vital events? If yes, what are the perceived benefits?
11. The success of registration of vital event to a great extent depends on awareness level of the communities? Were there any activities undertaken by you and other staffs of this office? If yes, please explain.
12. What challenge do you face in registering of vital events in your village/city?
13. What are your suggestions for further improving the level of registration of vital events?

**KII- Aganwadi Sevika**

1. Background characteristics

Age, Sex, Position and Years of experience

1. What is the role of the Aganwadi Sevika in birth/death registration? Who are the other stakeholders involved within the gram panchayat for birth and death registration process? What are their roles and responsibilities?
2. Is there any documentation required for registering the birth/death or is it an automated process? If yes, what are the documents required?
3. Is there any way to know the number of children within the gram panchayat whose birth registration was not done? If yes, what is that way and how frequently is the data updated? How is the issue of delayed registration addressed?
4. How is the information about the number of home deliveries collected? Is there any gap between the number of home deliveries reported and number of births registered? If yes, what are the possible reasons? How do you clear the backlog, if any?
5. What is the time gap between birth registration and issuance of birth certificate? What are the reasons for the gap and what could be done to reduce this gap?
6. What support do you receive from the DSO / BSS / GP/ CDPO for strengthened implementation of birth/death registration?
7. Have you received any training on birth/death registration? If yes, when was the training received and what was the content of the training? Do you feel there is any need for refresher training? If yes, what should be the content?
8. Are review meetings on birth/death registration held at the block / district/ Gram Panchayat/ CDPO level? If yes, what is the frequency of such meetings? What issues are generally discussed? Is there any feedback mechanism?
9. Do communities perceive any benefits of registering vital events, particularly in terms of access to government services? If yes, what are the perceived benefits?
10. The success of birth/death registration to a greater extent depends on the awareness level of the communities. Was there any IEC activities undertaken by you for birth/death registration? If yes, what were those?
11. What according to you are the other challenges in terms of birth/death registration in your gram panchayat?
12. What are your suggestions for further improving the level of birth/death registration in your area?

**FGD-Community Members**

1. Background characteristics

Age, Sex, Education level, Occupation, Residence (Rural/Urban), Duration of living in current residence (years)

1. Opinions and experiences of the public about death registration
2. Where and when did you register, when birth takes place in your household?
3. What is the process of registration and, what are the documents required to register birth?
4. Could you all please share your experiences in registering the birth event? Did you face any problems?
5. What do you think about the registration procedure in your area? (Simple or Complicated)? Why do you think so?)
6. Have you received birth certificate from the offices you applied at?
7. if no- why?
8. if yes- why?

**Death registration**

1. Do you think people in your area usually register deaths? if yes, where do they report?
2. What is the process and which documents are required for it?
3. Could you all please share your experiences in registering the death event? Did you face any problems?
4. What do you think about the registration procedure in your area? (Simple or Complicated? Why do you think so?)
5. Did you request death or burial certificate when the death event occurred in the household?
6. If no, why?
7. If yes, why?
8. Which circumstances do you think can encourage/discourage people registering deaths?
9. Tell me about your opinions on how registration of deaths can be improved in your area?
