# Supplementary material for: Performance barriers of Civil Registration System in Bihar: An exploratory study
Source: PLoS One. 2022 Jun 1;17(6):e0268832. doi: 10.1371/journal.pone.0268832 (PMC9159592; doi:10.1371/journal.pone.0268832)
Supplement: S1 Table — (DOCX) [file pone.0268832.s003.docx]

**S1 Table. Rate (%) of health and education indicators in the Bihar and India, 2015-2016.**

| **S. No.** | **Indicators** | **Bihar** | **India** |
| --- | --- | --- | --- |
| **1** | **Total population (2016) (million)** | 113^a^ | 1324 |
| **2** | **SCs Population (%)** | 15.91 | 16.63 |
| **3** | **STs Population (%)** | 1.28 | 8.60 |
| **4** | **Proportion Hindus (%)** | 82.69 | 79.80 |
| **5** | **Proportion Muslims (%)** | 16.87 | 14.23 |
| **6** | **Total Fertility Rate** | 3.14 | 2.18 |
| **7** | **Crude Birth Rate** | 27.00 | 19.00 |
| **8** | **Under-five mortality rate (per 1000 live births)** | 58.00 | 50.00 |
| **9** | **IMR (per 1000 live births)** | 48.00 | 41.00 |
| **10** | **Twelve or higher years of schooling among household populations age six and above (%)** | 6.80 | 13.70 |
| **11** | **Children who received immunisation from anganwadi sevika (%)** | 41.10 | 39.80 |
| **12** | **Children who received health check-ups from anganwadi sevika (%)** | 32.80 | 31.20 |
| **13** | **Institutional birth (%)** | 64.00 | 79.00 |
| **14** | **Birth registration level (%)** | 74.70 | 84.40 |
| **15** | **Death registration level (%)** | 37.10 | 69.30 |
| **16** | **Net State Domestic Product Per Capita (2018-19) (US$)** | 1964.00 | 640.00 |

Data Source:-**For Health Indicators**: NFHS, 2015-16**; For SCs, STs, Religion**: Census 2011**; For Net State Domestic Product Per Capita**: MOSPI**; For Population**: United Nations_._^a^ Estimated population of Bihar (2016) = Estimated population of India (UN, 2016) * proportion of the population of Bihar (8.57%) to India (census 2011); **Birth &** **Death Registration Level**: ORGI, 2018
