# Supplementary material for: Performance barriers of Civil Registration System in Bihar: An exploratory study
Source: PLoS One. 2022 Jun 1;17(6):e0268832. doi: 10.1371/journal.pone.0268832 (PMC9159592; doi:10.1371/journal.pone.0268832)
Supplement: Attachment — Submitted filename: PONE-D-21-33029_R1.pdf [file pone.0268832.s010.pdf]

PLOS ONE

Performance barriers of Civil Registration System in Bihar: An exploratory study

--Manuscript Draft--

**Manuscript Number:**	PONE-D-21-33029R1
**Article Type:**	Research Article
**Full Title:**	Performance barriers of Civil Registration System in Bihar: An exploratory study
**Short Title:**	Civil Registration System in Bihar
**Corresponding Author:**	Nandita Saikia, PhD<br>International Institute for Population Sciences<br>Mumbai, INDIA
**Keywords:**	Bihar; Civil Registration System; India, Death, Mortality
**Abstract:**	Objectives: Vital statistics generated by the Civil Registration System (CRS) are essential for developing healthcare interventions at all administrative levels. Among all India's states, Bihar showed a lower recording of birth and death in 2019. This study investigates CRS's performance barriers from the perspective of CRS staff and community members in Bihar.<br><br>Methods: We conducted a primary qualitative survey in the two districts of Bihar during February-March 2020 with CRS staff (n=15) and community members (n=90). We purposively selected the Patna and Vaishali districts of Bihar for the survey. Thematic analysis was done to identify the pattern across the data using the Atlas-ti software.<br><br>Results: Most participants showed a good understanding of registration procedures and birth and death registration benefits. The perceived need for death registration is lower than birth registration. While birth registration was higher among female children than male children, death registration was lower among females. We found that most participants did not report children or adult female death due to lack of financial or property-related benefits. Death registration was not mandatory for burial or cremation permits. Most participants faced challenges in reporting birth and death due to poor delivery of services at the registration centres, higher indirect opportunity cost, and demand of bribes by the CRS staff for providing certificates. We found a lack of adequate investment, a dedicated staff shortage, and limited computer and internet services at the registration centers.<br><br>Conclusions: Poor data on birth and death registration could lead decision-makers to target health services inappropriately. Strengthening health institutions' linkage with the registration centres, mobile registration in far-flung areas and regular CRS staff training could increase death registration levels. An adequate awareness campaign on the benefits of birth and death registration is required to increase the reporting of vital events.
**Order of Authors:**	Krishna Kumar, MPhil
	Nandita Saikia, PhD
	Nadia Diamond-smith, PhD
**Response to Reviewers:**	Reply to respected reviewers.<br><br>Reviewer #1: This is a good piece of scientific work addressing a very important yet neglected issue. Good efforts have been put together to develop this manuscript though it needs some major revisions.<br>Major points:<br><br>1. Line Nos 121-129: Please make the study rationale more clear<br>Reply- We have revised the manuscript in light of your suggestion. Please see line no. 121-130.<br><br>2. Figure 1:<br>a. How are the deaths at 'home' in 'urban' areas registered? Missing in the diagram.

Reply- We have revised the diagram in light of your suggestion. Please see page no.5, fig. 1

b. How are the deaths in 'hospitals' in rural areas registered? missing in the diagram.
Reply- We have revised the diagram in light of your suggestions. Deaths in Hospitals are registered by the head of the institution (Chief Medical Officer). Please see page no.5, fig 1.

c. Is ASHA/ANM playing any role in death registration as being currently used in Punjab and Haryana? if yes, then mention, please. Who are the designated key informants to whom family members can report FIR of an event in both urban and rural areas?
Reply- In Bihar, ASHA/ANM does not play any role in birth and death registration. We have revised the manuscript now. Please see page no. 5, fig 1

d. At the district and state level, which department is responsible for births and deaths registration? Is it the Health department like in Haryana and Punjab? These points are not coming out explicitly from the diagram itself!!
Reply- Registrar at Nagar Nigam, District registrar office, and Chief medical officer of district-level hospitals are mainly responsible for birth and death registration. At the state level, Chief registrar, Joint Chief Registrar, and Dy. Chief Registrar (Vital Statistics) is responsible for birth and death registration. We have revised fig 3. (Page no. 7)

3. Please mention three things in each box of Figure 3: Designation of the person under CRS, name of the department the person holds, and his/her designation in that department. Example: Like you have mentioned Chief Registrar is the Director of Statistics and Evaluation but of which department???? Please elaborate
Reply- Chief Registrar is the Director of the Directorate of Statistics and Evaluation belonging to the Planning and Development Department in Bihar. We have revised fig 3 in light of your suggestion. Please see fig3, Page no. 7

4. Did you consider the levels of birth and death registration of district and block as selection criteria for sampling units because that might give a better picture of reasons.
Reply- We observed district-level death registration level as selection criteria in addition to urbanization rate. We purposively selected a district with higher death registration and a district with lower death registration. Furthermore, four blocks are chosen randomly from each district. We have revised the write-up now. Please see line no. 153-159.

5. The methodology is indicating that the study was conducted among the rural aspect of CRS as none of the study participants represented the urban population or municipal corporation. This has undermined the study findings to rural areas only. I feel you need to explain this as a study limitation and provide a valid reason for not including the urban representation.
Reply- We also included considerable study participants from urban areas. We have presented the methodology more clearly now. Please see line no. 170-177.
6. In the methodology section, it's important to mention the levels of birth and death registrations of the two districts you have selected. and compare it with other districts.
Reply- We have included the death registration level of two selected districts (Patna and Vaishali) in methodology (lines no. 157 & 159). We have provided a supplementary table showing birth and death registration levels by the districts of Bihar. Please see supplementary file S3 Table.

Minor revisions:
1. Please use one terminology throughout either CRS or CRVS in the introduction section
Reply- We have revised the manuscript in light of your valuable suggestions. We are using CRS throughout the manuscript.

2. Expand ORGI in the caption of Figure 1
Reply- We have revised the manuscript. We have written a different title now. ” Fig-1 Flow chart of birth and death registration in Bihar” (Please see page no. 5)

Powered by Editorial Manager® and ProduXion Manager® from Aries Systems Corporation

3. Please correct the spelling of 'Anganwadi sevika' throughout the manuscript
Reply- We have corrected the spelling of Anganwadi sevika in the revised manuscript.

4. Mention in the data collection part of the methodology section about the total number of FGDs done
Reply- We have included the total number of FGD done in the revised manuscript. Please see line no.- 183

5. Mention in the data collection part of the methodology section about the total number of KII done
Reply- We have included the total number of KII done in the revised manuscript. Please see line no. 183

6. Line nos. 290-93 not clear
Reply- We have revised the manuscript now. Please see line no. 248-253

7. Please provide supporting data for statement no. 528-529
Reply- We have included the supporting data now. Please see line no. 505

8. Please provide supporting data for statement no. 338-39
Reply- We have found lower reporting for death, particularly, infant and female death, in Bihar. We provided the transcript along with this manuscript. A study also showed death registration coverage is poor in Bihar (Reference no. 38 in the manuscript)

9. Please add references for lines 182-186
Reply- We have included a supplementary table (S3 table) in support of lines 182-186.

10. In line no. 556-57, you have mentioned that birth and death registration is not being done at sub-center or village level? Is it not community-based as of now? Review the lines
Reply- In rural areas, birth and death occur at home are registered by Anganwadi sevika within 21 days of the event. In urban areas, birth and death occur at home is registered by registrar at municipal corporation and block offices. Institutional birth and death is registered by designated health staff at health centers within 21 days of events. Please see fig 1, page no. 5. We have revised the manuscript now.

11. You have mentioned in line 567 that all PHCs are not linked to CRS. Is this statement true? Please review.
Reply- We have found many PHC are not linked to CRS due to lack of adequate logistics and lack of dedicated staff or negligence by the medical officer. A recent paper also showed registration are missed in PHC due to negligence (Kumar et al., 2021)

Reviewer #2: Review: Performance barriers of Civil Registration System in Bihar: An exploratory study

This is an interesting exploratory study, relying on interviews with government officials and rural residents, which documents the functioning of the civil registration system in Bihar before the pandemic. Given the importance of understanding and improving the civil registration system, such studies are urgently needed. I felt that the study should have been more in-depth. Interviews with CRS officials and with community members could have been more detailed, and the description of the overall state of the CRS in Bihar, despite the authors' attempts, is still quite incomplete. However, given the paucity of scholarship on the civil registration system in India, specially of a qualitative nature, this paper is welcome. I have some minor suggestions, which I hope would be helpful for the authors in revising their work.

1. The authors motivate their paper saying that it is important to study the civil registration system in Bihar because of its poor development indicators in general, and the fact that its CRS is estimated to record the lowest proportion of deaths and births. However, although the authors provide some hints at why Bihar's CRS performs so poorly, we do not get an explicit discussion of why precisely Bihar lacks even other northern Indian states, such as Madhya Pradesh or Rajasthan, which have higher registration coverage than Bihar. In this, the state of Bihar and Uttar Pradesh are way

behind all the other states in India, and it is not clear why this is the case. To truly understand this, a comparative design is needed. Perhaps the case is that the lack of linkages between health systems and the CRS which the authors document, or the lack of computerization in Bihar explains why it lags behind other Indian states (even similarly poor ones). My suggestion to the authors is to consider this line of thinking carefully. They can see what the extant literature says about other poor Indian states (they may find looking at the annual CRS reports at the state level and the national level useful here) about CRS being so poor in Bihar. They should also note some lines of thinking for future researchers in the discussion section, as well as perhaps a limitation that they have not adequately addressed these questions.
Reply- There are limited studies on CRS at state levels, particularly in Bihar. We could not locate any study examining why CRS is poor in Bihar. Although we welcome the reviewer's comment on a comparative picture of Bihar/UP with MP or Rajasthan, it is beyond the scope of the current study. Therefore, a comparative study cannot be done for the present study as it collected information of Bihar only. We may explore this research gap in future studies. We have included these lines in the limitation section of this manuscript. Please see line no. 560-561.

2. Related to this point, we also don't get a sense of the changes in the Civil Registration System before the pandemic in Bihar. Existing patterns, both from the data that has been accessed by journalists during the pandemic period (for instance see https://www.indiacovidmapping.org/reports/mortality/BiharFactsheet.pdf), as well as annual estimates before the pandemic suggest improvements in the Civil Registration System in Bihar. How did these improvements came about? If the authors have information on this, it would be extremely valuable. Similarly, what happened to Bihar's civil registration system during the pandemic? Answering this likely requires fresh data collection. If that is not feasible, then that should be noted in the discussion. If it is possible to call CRS staff on phones and ask some of these questions, then that would be quite valuable. If this is not possible, then perhaps the authors can consider this as a future research endeavor.
Reply- We have included information regarding development of CRS in Bihar before pandemic. Also, we added what happen to CRS in Bihar during the pandemic in the revised manuscript. Please see line no. 452-455.& 459-466.

3. At a large number of places, I felt that the authors have policy recommendations that were not supported by the information they present. In some cases, their recommendations can actually be harmful. For instance, the authors recommend "mandatory burial or cremation permits." Instead of improving registration, I can see how this kind of procedure would cause even more problems in a place like Bihar. For instance, people will have to wait to get a death registered and could then only approach burial or cremation grounds? Instead of this, what would work well is a "notification" and "proof" system: burial grounds can notify CRS authorities about a death, and the family could have a slip which would help as a proof of death. These linkages exist in other states, especially with healthcare facilities. Public hospitals can in fact issue death certificates in many states, and private hospitals issue a hospital death certificate (or birth certificate) which can be taken to a registrar to get a death registered.

I was also uncomfortable with the recommendation of fines (lines 533-534). First, the authors already say that there is a fine of Rs. 10 to register a death after 21 days. Worse, the procedure described here to register a death after 21 days looks like a nightmare, with visits to far off offices. More fines when clearly it is the government system that is lacking is a bit counterproductive. The authors should remove this recommendation. In any case, this does not follow from the data presented by the authors.
Reply- We have revised the manuscript in light of your valuable suggestions. We have deleted mandatory registration burial or cremation and fine for late registration.

4. I found the sentence "there was no strict implementation of the birth and death registration law" vague. Which aspects of the law were not strictly implemented, and how was this ascertained?
Reply- We found health institution does delayed registration; however, it is not allowed as per official guidelines. These event should be registered by Block Development

	Officer or District Statistical Officer in such cases. A similar problems were also shown by a previous literature (Kumar et al; 2021).We have included this sentence in revised manuscript. Please see line no. 433-434.<br><br>5. I was not convinced by the literature cited that “lack of birth registration is associated with school dropout …” and so on. It is quite likely that this is just selection, for instance poor children are more likely to drop out and poor children are less likely to have had their births registered. I would suggest dropping this citation.<br>Reply-We tried to show the association of unregistered birth and school dropout. We don’t intend to show absence of birth certificate is a causal factor of the school drop-out. However, we have reframed the sentence now. Please see line no. 91-92<br><br>6. The authors cite the CRS report to report that 7 percent of births and 8 percent of deaths were not registered in 2019. Although this is an official estimate, it is likely an over-estimate. There are multiple reasons for this, among them the fact that the 2019 CRS deaths in this report are compared to 2018 SRS. Other reasons include the fact that the SRS may under-estimate the crude death rate. This line of thinking is explored in detail here (appendix 1:<br>https://www.medrxiv.org/content/10.1101/2021.09.30.21264376v1). I leave it to the authors to decide how best to convey the uncertainty in the true rate of registration completion. Perhaps the authors can say “according to official estimates …”.<br>Reply- We have written “According to official statistics”, line no. 95-96.<br><br>7. In figure 1, which provides the organization of the CRS, I was surprised that the Panchayat was not mentioned anywhere in the hierarchy. In figure 3, the panchayat sevak is mentioned as a registrar. In some other northern states, such as Madhya Pradesh, the bulk of rural death and birth registration happens at the Panchayat level. Is it the case that this does not happen in Bihar? In Madhya Pradesh, I have seen functioning computers in Panchayat offices which can register deaths. In which case, this may be the reason why death registration is so poor in Bihar – the lack of panchayat registration. The authors could perhaps explore this more, and correct figure 1 if necessary. If the panchayat is not a functional place to register deaths, then this is actually an important policy suggestion, to improve registration and computerization at the Panchayat level.<br>Reply- Panchayat secretary office registers birth and death after 21 days of the occurrence of event in rural areas in Bihar. I have revised the fig 1. Please see page no. 5<br><br>8. Figure 1 claims that an annual report at the “state level is published by the chief registrar” and also that “Annual report at India and state level is published by ORGI”. This is confusing, and likely the latter sentence needs modification.<br>Reply- Annual report at India level is published by ORGI, New Delhi. In Bihar, state level report is published by Directorate of Economic and Statistics, Department of planning and development. We have changed the fig 1 now. (Please see page no .5)<br><br>9. I thought that the bit about people not reporting infant deaths because it is considered a sin of parents in previous birth was quite revealing. The state government should consider potentially challenging this belief, and this also is a policy recommendation that flows from the findings from the paper. In addition, given the levels of awareness that the authors document, perhaps more information campaigns are also worth recommending.<br>Reply- We have revised the manuscript in light of suggestions. Please see line no. 544-45<br><br>10. Similarly, I was surprised by the sentence that “Public facilities record all birth and death but don't update on the CTS portal until birth and death certificates are requested by family members.” The authors do not present evidence for it. This is quite important, and also deserves to have highlighting from the perspective of policy recommendation that the paper generates.<br>Reply- We observed health staff don't update registered data from registers to the CRS portal until birth and death certificates are requested by family members. A study documented a similar problem (Reference no. 38). We recommended for proper monitoring of all registration units. Please see line no. 548

Powered by Editorial Manager® and ProduXion Manager® from Aries Systems Corporation

11. In line 536: the authors say the CRS form is complex. Perhaps they should elaborate more? I do not agree with all the things the CRS form asks (for instance, whether the person smoked or drank alcohol is not collected by most other countries) but I didn't have the impression that it was very complex. If the authors think it is complex, I would like to see an explanation why. Otherwise, I would like this sentence to be reformulated.
Reply- We have removed this sentence in the revised manuscript.

Reviewer #3: I am reviewing this paper from a non-expert perspective - I have limited knowledge of the civil registration system itself, and the specific issues faced in Bihar.

The paper clearly explores an important issue, namely barriers to civil registration in Bihar. The importance of strengthening civil registration has been highlighted by the pandemic, making this study timely. The methodology - interviews with key informants and members of the public - seems appropriate, and the paper includes a number of interesting insights about these barriers at both administrative and cultural levels.

I have a few comments for the authors to consider:

1. Quantitative data. Although this is a qualitative study, it would still be helpful to have some summary quantitative data. At least: amongst the focus group participants in Patna how many births and how many deaths were reported; and how many of these were registered. Plus the same for Vaishali. This is not to draw any quantitative conclusions, but merely to understand the overall sample.

In fact, some quantitative data is presented, but too unclearly. "A few participants (n=15) reported death at the registration centres situated at cemeteries or burial places" - this sentence needs a denominator: 15 out of how many? In sentences such as "Besides, two-fifth of the study participants reported the death of their household members at the block or municipality offices." it would be better to present the actual numbers of how many deaths were reported, and out of these how many at block or municipality offices.

The same comments apply at several other places in the text, for example: "Nearly one-sixth of the total participants were unaware of the death registration procedures and benefits associated with death registration. A higher number of participants from Patna applied for birth and death registration of their household members than participants from Vaishali. Rural and urban differentials in birth and death registration were found." In every case numbers, with denominators, would be better.

"However, a subset of participants (n=10) did not receive a birth certificate for their child at all." Again, a denominator is needed. Also, not clear: were these people who applied for, but never received, the birth certicificate; or who never applied for the certificate?
Reply- Dear reviewer, we have not explored categorically the number of registered births and deaths, in this study. However, we have shown how many participants registered birth and death of their family members. Besides, we have included denominator in the revised manuscript in light of your suggestions.

2. Context and history of death registration in the state. The authors mention inconsistency in death registration in Bihar - the lack of a clear trend (up to 2019 at least). Do they have any insights as to why there is so much year-to-year variation in the estimated levels of death registration? Are the SRS-based estimates of coverage reliable in their view? What are the factors which could cause a significant drop in registration from one year to the next? If there were changes in the systems in place which affected trends in registration, then this would be interesting to know. Did the interviews provide any hints?
Reply. Our primary study revealed that birth and death registration was decreased during 2013 and 2014 due to majority of CRS staff are engaged in Bihar Assembly election duty. Thereafter, there has been continuous increase in birth and death registration due to introduction of digital registration using CRS portal and awareness campaign.

3. Poverty and marginalisation. I find these sentences problematic: "Lack of birth

Powered by Editorial Manager® and ProduXion Manager® from Aries Systems Corporation

registration is associated with increased school dropout, child trafficking, and child labor [1,12]. The child mortality rate is higher among unregistered children [13]." It is very likely that the common factor behind these associations is poverty and marginalisation - the sentences are not formally wrong, but suggest the lack of birth registration could be a causal factor behind, say, child trafficking or infant mortality. It seems important to be more clear here.

This raises an important point: the authors really should discuss how factors such as caste and poverty are associated with levels of registration. Are there studies on this topic, relevant to Bihar? If there is a relevant literature, then it should be referenced and discussed at least briefly. It would be interesting to know whether there are examples of other states where poverty and marginalisation are comparable to Bihar, but civil registration is higher and more stable, and if the authors have some insight about why. If many people in Bihar perceive little benefit in reporting deaths, then why is this different in other comparable states? Are the practical barriers - loss of time and income, difficulty, etc., different in other states?

Although the sample may be too small to understand how attitudes to registration vary by caste/class there still seem to be some hints in the data (e.g. not registering infant deaths - "sin of previous birth"). It seems that the authors did not collect demographic data on caste/ occupation/ socioeconomic status, etc? Perhaps the authors should mention this as a limitation of the study. It would still be interesting if they have any insights from this work or the work of others on how these factors might affect access to registration or attitudes to registration.
Reply- We have shown association in the sentence “Lack of birth registration is associated with increased school dropout, child trafficking, and child labor”, not the causal factor. Moreover, we have reframed this sentence in the revised manuscript “A low birth registration is associated with increased school dropout, child trafficking, and child labor”. We did not collect socio-economic data in this study. Also, comparison study with other similar state cannot be done at present due to limited literature. We have mention this sentence in limitation section of this study. Please see line no. -559-560

4. Recommendations. A number of the recommendations for how to improve vital registration are made by the authors, and many of these could be useful. But it is not really clear where responsibility lies for the failings. After statements like "the officers did not take any steps for improving the registration level in recent times, particularly death registration." could the authors indicate what the officers could have done? Also perhaps there could be more discussion of resources. A "lack of adequate investment" is mentioned in the abstract, and later the key informants "...suggested that adequate funds should be provided for the smooth functioning of the system." What is the resourcing of the CRS in Bihar? Did the interviews provide insight into where such funds should go (more staff? better pay? better training? more registration offices?) Here, again, some comparison with other states would be interesting.

Reply- We have revised the manuscript in light of the suggestions. However, there is limited study on CRS in India, so a comparative study cannot be done at this stage. We will explore this issue in a future study. We have included some suggestions on the role of authority in the recommendation section of this manuscript. Please see line no. 543-551

5. Minor points

"Epidemiological purposes" are mentioned in the introduction - the authors could say more about how, in the context of the current pandemic, there has been an urgent neall-causel cause mortality data on account of weak official surveillance of COVID mortality. Any comments on uncertainties around pandemic mortality in Bihar and in comparable states would help to highlight the current importance of this study.

Reply- We have revised the manuscript in view of suggestions. Please see line no. 452-455

In Table S1, the fertility rates for Bihar and India appear to be exchanged.
Reply- We have cored the Table S1 .Please see supplementary table (S1 table).

	Table S1 should perhaps include the estimated death registration level based on SRS and/or NFHS?<br>Reply- We have included the official estimate of death registration level (Reference- Office of Registrar General of India (ORGI), Report, 2018). ORGI also use SRS death rate for calculating death registration level.<br><br>I would suggest the authors carefully reread the manuscript for typographical errors.<br>Reply- We have revised the manuscript now.
**Additional Information:**	
**Question**	**Response**
**Financial Disclosure**<br><br>Enter a financial disclosure statement that describes the sources of funding for the work included in this submission. Review the submission guidelines for detailed requirements. View published research articles from *PLOS ONE* for specific examples.<br><br>This statement is required for submission and **will appear in the published article** if the submission is accepted. Please make sure it is accurate.<br><br>**Unfunded studies**<br>Enter: *The author(s) received no specific funding for this work.*<br><br>**Funded studies**<br>Enter a statement with the following details:<br>• Initials of the authors who received each award<br>• Grant numbers awarded to each author<br>• The full name of each funder<br>• URL of each funder website<br>• Did the sponsors or funders play any role in the study design, data collection and analysis, decision to publish, or preparation of the manuscript?<br>• **NO** - Include this sentence at the end of your statement: *The funders had no role in study design, data collection and analysis, decision to publish, or preparation of the manuscript.*<br>• **YES** - Specify the role(s) played.<br><br>* typeset	The author(s) received no specific funding for this work.
**Competing Interests**<br><br>Use the instructions below to enter a	The authors have declared that no competing interests exist.

competing interest statement for this submission. On behalf of all authors, disclose any competing interests that could be perceived to bias this work—acknowledging all financial support and any other relevant financial or non-financial competing interests.<br><br>This statement is required for submission and **will appear in the published article** if the submission is accepted. Please make sure it is accurate and that any funding sources listed in your Funding Information later in the submission form are also declared in your Financial Disclosure statement.<br><br>View published research articles from *PLOS ONE* for specific examples.<br><br>**NO authors have competing interests**<br><br>Enter: *The authors have declared that no competing interests exist.*<br><br>**Authors with competing interests**<br><br>Enter competing interest details beginning with this statement:<br><br>*I have read the journal's policy and the authors of this manuscript have the following competing interests: [insert competing interests here]*<br><br>* typeset	
**Ethics Statement**<br><br>Enter an ethics statement for this submission. This statement is required if the study involved:<br><br>• Human participants<br>• Human specimens or tissue<br>• Vertebrate animals or cephalopods<br>• Vertebrate embryos or tissues<br>• Field research	The study protocols have been reviewed and approved by the institutional ethical review board at the Jawaharlal Nehru University (Ref. No. 2019/Student/235).

Powered by Editorial Manager® and ProduXion Manager® from Aries Systems Corporation

Write "N/A" if the submission does not require an ethics statement.

General guidance is provided below. Consult the submission guidelines for detailed instructions. **Make sure that all information entered here is included in the Methods section of the manuscript.**

Format for specific study types

Human Subject Research (involving human participants and/or tissue)

- Give the name of the institutional review board or ethics committee that approved the study
- Include the approval number and/or a statement indicating approval of this research
- Indicate the form of consent obtained (written/oral) or the reason that consent was not obtained (e.g. the data were analyzed anonymously)

Animal Research (involving vertebrate animals, embryos or tissues)

- Provide the name of the Institutional Animal Care and Use Committee (IACUC) or other relevant ethics board that reviewed the study protocol, and indicate whether they approved this research or granted a formal waiver of ethical approval
- Include an approval number if one was obtained
- If the study involved *non-human primates*, add *additional details* about animal welfare and steps taken to ameliorate suffering
- If anesthesia, euthanasia, or any kind of animal sacrifice is part of the study, include briefly which substances and/or methods were applied

Field Research

Include the following details if this study involves the collection of plant, animal, or other materials from a natural setting:

- Field permit number
- Name of the institution or relevant body that granted permission

Powered by Editorial Manager® and ProduXion Manager® from Aries Systems Corporation

**Data Availability**<br><br>Authors are required to make all data underlying the findings described fully available, without restriction, and from the time of publication. PLOS allows rare exceptions to address legal and ethical concerns. See the PLOS Data Policy and FAQ for detailed information.<br><br>A Data Availability Statement describing where the data can be found is required at submission. Your answers to this question constitute the Data Availability Statement and **will be published in the article**, if accepted.<br><br>**Important:** Stating 'data available on request from the author' is not sufficient. If your data are only available upon request, select 'No' for the first question and explain your exceptional situation in the text box.<br><br>Do the authors confirm that all data underlying the findings described in their manuscript are fully available without restriction?	Yes - all data are fully available without restriction
**Describe where the data may be found in full sentences. If you are copying our sample text, replace any instances of XXX with the appropriate details.**<br><br>• If the data are **held or will be held in a public repository**, include URLs, accession numbers or DOIs. If this information will only be available after acceptance, indicate this by ticking the box below. For example: *All XXX files are available from the XXX database (accession number(s) XXX, XXX.*).<br>• If the data are all contained **within the manuscript and/or Supporting Information files**, enter the following: *All relevant data are within the manuscript and its Supporting Information files.*<br>• If neither of these applies but you are able to provide **details of access elsewhere**, with or without limitations, please do so. For example:	All relevant data are within the manuscript and its Supporting Information files.

Data cannot be shared publicly because of [XXX]. Data are available from the XXX Institutional Data Access / Ethics Committee (contact via XXX) for researchers who meet the criteria for access to confidential data.<br><br>*The data underlying the results presented in the study are available from (include the name of the third party and contact information or URL).*<br>• This text is appropriate if the data are owned by a third party and authors do not have permission to share the data.<br><br>* typeset	
Additional data availability information:	Tick here if the URLs/accession numbers/DOIs will be available only after acceptance of the manuscript for publication so that we can ensure their inclusion before publication.

Powered by Editorial Manager® and ProduXion Manager® from Aries Systems Corporation

Click here to access/download;Manuscript;Manuscript.docx

Performance barriers of Civil Registration System in Bihar: An exploratory study

Krishna Kumar[1], Nandita Saikia[2*], Nadia Diamond-smith[3]

[1]Centre for the Study of Regional Development, School of Social Sciences III, Jawaharlal Nehru University, New Delhi, India 110067

Email: krishhna94@gmail.com

ORCID iD: http://orcid.org/0000-0002-6822-900X

[2]Department of Public Health and Mortality Studies, International Institute for Population Sciences, Deonar, Mumbai-400088

Email: nanditasts@gmail.com

ORCID iD: https://orcid.org/0000-0001-6735-6157

[3]Epidemiology and Biostatistics Department, University of California, San Francisco CA 94158

Email: Nadia.Diamond-Smith@ucsf.edu

ORCID iD: https://orcid.org/0000-0002-8711-3029

***Corresponding author:**

Nandita Saikia

Email: nanditasts@gmail.com

Running Title: Civil Registration System in Bihar

Word Count: 6636

Abstract

Objectives: Vital statistics generated by the Civil Registration System (CRS) are essential for developing healthcare interventions at all administrative levels. Among all India's states, Bihar showed a lower recording of birth and death in 2019. This study investigates CRS's performance barriers from the perspective of CRS staff and community members in Bihar.

Methods: We conducted a primary qualitative survey in the two districts of Bihar during February-March 2020 with CRS staff (n=15) and community members (n=90). We purposively selected the Patna and Vaishali districts of Bihar for the survey. Thematic analysis was done to identify the pattern across the data using the Atlas-ti software.

Results: Most participants showed a good understanding of registration procedures and birth and death registration benefits. The perceived need for death registration is lower than birth registration. While birth registration was higher among female children than male children, death registration was lower among females. We found that most participants did not report children or adult female death due to lack of financial or property-related benefits. Death registration was not mandatory for burial or cremation permits. Most participants faced challenges in reporting birth and death due to poor delivery of services at the registration centres, higher indirect opportunity cost, and demand of bribes by the CRS staff for providing certificates. We found a lack of adequate investment, a dedicated staff shortage, and limited computer and internet services at the registration centers.

Conclusions: Poor data on birth and death registration could lead decision-makers to target health services inappropriately. Strengthening health institutions' linkage with the registration centres, mobile registration in far-flung areas and regular CRS staff training could increase death registration levels. An adequate awareness campaign on the benefits of birth and death registration is required to increase the reporting of vital events.

Introduction

A Civil Registration System (CRS) is a permanent, continuous, compulsory, and universal recording of vital events with the legal requirements [1]. The Civil Registration Vital Statistics is exact and actual data, certified by registering authority, and legally acceptable [2]. A birth certificate documents information such as age, place of birth, and family background [3]. Besides official documentation of a child's birth, it facilitates government-aided essential services such as education, health facilities, and social security [4-6]. CRS also provides timely and reliable vital statistics. Health officials and policymakers mainly use CRS data to track fertility, mortality, and epidemiological patterns at all administrative levels. United Nations documented that a functional CRS is the best source of information on vital events for administrative, demographic, and epidemiological purposes [7].

Despite government agencies and UNICEF's effort to universalize birth registration globally, about 166 million children under age five and 40 million infants were not officially documented [3]. According to UNICEF's report (2019), about 77 million children under age five do not have a birth certificate in South Asia. There has been a significant disparity among countries in terms of birth registration. Low or middle-income countries showed more unsatisfactory performance in registering the child's birth. Mortality statistics are also essential for identifying health risks and evaluating health programs. In many countries, the completeness of death registration is lower than birth registration. World Health Organisation (WHO) recorded about the two-thirds of global deaths were not registered. Besides, most WHO members obtained incomplete data on mortality and the cause of death [8]. Another study showed that administrative challenges, insufficient technical capacities, and unawareness are significant challenges in death registration [9]. Accurate recording of death's cause is challenging in lower and middle-income countries [10,11].

In India, the Births, Deaths, and Marriage Registration (RBD) Act, enacted in 1886, suggested voluntary registration of births and deaths. However, it was not uniformly implemented across India. After independence, India's Government introduced the Registration of Births and Deaths Act in 1969, which mandates registration of all births and deaths within 21 days [2]. A low birth registration is associated with increased school dropout, child trafficking, and child labor [1,12]. A higher child mortality rate is associated with higher level of unregistered children [13]. A

lower death registration limits medical research and reliable mortality estimates [2,14]. Despite India's mandatory birth and death registration law, according to official statistics, nearly 7 percent of births and 8 percent of deaths were not registered in India in 2019 [2]. Further, there are disparities at the state and district level regarding coverage and completeness of birth and death registration. In India, seventy percent of the districts have more than a million population each; lower death registration coverage at the district level precludes estimating health indicators with precision [15]. Universal civil registration in the interest of sound policymaking and its implementation helps monitor the SDGs goals such as promoting healthy lives and well-being, reducing mortality rate and legal identity for all [16, 17].

Bihar is one of the least developed and third most populated state of India. A comparison table of demographic and health indicators of Bihar and India is shown in S1 Table. The total population of Bihar is 113 million (which is equivalent to the population size of Ethiopia, the world's 12th populous country), constituting 8.57% of India's population. According to the National Family Health Survey, 2015-16, Bihar has the highest total fertility rate (3.14) in India. The infant death rate and under-five mortality in Bihar are 48.1 and 58.1 per thousand live births. Education attainment is quite low, with only 14 percent of the household population of eighteen and above completed 12 or more years of schooling. Among the children, age 0-6 years, 41 percent and 33 percent received immunisation and health check-up from an anganwadi sevika. The percentage of institutional birth is lower in Bihar (64%) as compared with India level (79%) [18]. In Bihar, birth and death registration levels are much lower than the national average. However, there has been an increase in birth registration levels in Bihar from 64% in 2014 to 89% in 2019 [2,19]. On the other hand, the death registration level was lower (52%) in Bihar compared with other Indian states in 2019 [2]. Additionally, Bihar showed inconsistency in death registration level from 43% in 2017 to 35% in 2018 and further, the level increased to 52 percent in 2019 [2,20,21].

There has been increased research around the impact of under-registration, but existing studies focused on the need and benefits associated with functional registration systems [6,22,23]. Recent studies primarily focused on birth and death registration levels, trends and estimation of death registration level using indirect methods [24,25,26]. However, such studies did not answer why death registration is lower in some states of India. While media reports and public health

experts highlighted the importance of up-to-date vital statistics for initiating healthcare intervention during a health emergency, peer-reviewed literature investigating motivations and barriers to birth and death registration in India is limited. Previous studies suggested qualitative studies in the states or districts where a lower registration level was recorded to explore the contextual determinants of under registration [7,9,27]. This study investigates the performance barriers of the CRS in Bihar, a state with the lower level of birth and death registration. This study provided crucial inputs to reform administrative issues for an effective CRS in Bihar.

Materials and Methods

We conducted a primary qualitative survey in the two districts of Bihar during February-March 2020 with CRS staff (n=15) and community members (n=90). Fig 1 shows the framework of the operation of CRS in Bihar.

Fig 1. Flow chart of birth and death registration in Bihar

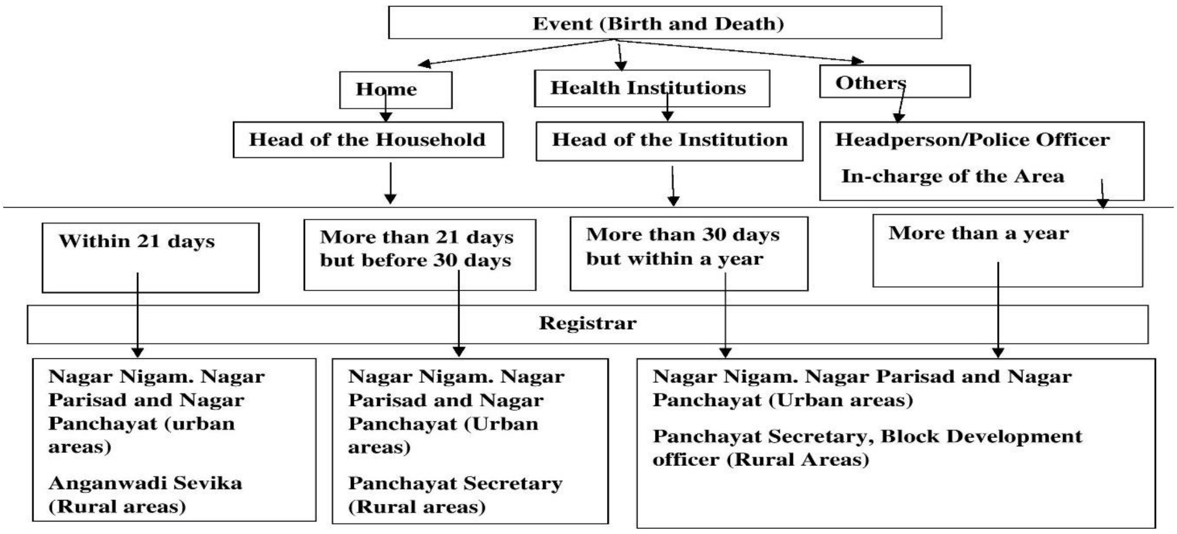

CRS annual report, 2020. Department of Planning and Development, Bihar, 2021

Note:- In case of birth and death registration after 30 days but within 1 year, registration is done after written permission of the prescribed authority and on the submission of an affidavit made before a notary public and payment of a late fee of rupee five (US$ 0.07)

In case of registration of birth and death after an year, an order is required by a magistrate of the first class (Block Development Officer) after verifying the correctness of the event and payment a fee of rupee ten (US$ 0.14)

Study design and sample

Fifteen CRS staff and ninety community members participated in the survey. Out of 38 districts in Bihar, we purposively chose two districts Patna and Vaishali, from which we randomly selected four blocks from each district. Fig 2 shows the study area. We chose Patna and Vaishali districts to investigate how registration practices differ in two different contexts. These districts represent two different urbanization contexts and registration levels: Patna being the most urbanized (43%) and Vaishali the least urbanised district (7%) of Bihar. Patna has better health care facilities, a better road network, CRS offices equipped with modern technologies, death registration level (61%) and a higher literate population (71%). On the other hand, Vaishali has a poor road network and transport facilities, lower access to public and private health institutions, death registration level (45%) and a lower proportion of the literate population (67%) See S3 table (sheet 3). We selected Patna Sadar, Sampatchak, Phulwarisarif, and Danapur blocks from Patna, and Hajipur, Vaishali, Desri, and Raghopur blocks from Vaishali. 4 out of total 22 blocks of Patna were randomly selected using a RANDBETWEEN function in excel. Similarly, 4 out of 16 blocks of Vaishali were selected (see S2 Table). We also showed estimated birth and death registration level by districts of Bihar (See S3 Table).

INSERT Fig 2 HERE

Source- Authors generated the map using GIS

Study participants

Block Statistical Officer (BSO), a Medical Officer, a Panchayat Chief or Ward Parsad (local leader), an Anganwadi Sevika and a registrar at Nagar Nigam from each block were chosen for the key informant interview (KII). An anganwadi sevika is a community health worker from the local village who delivers services to pregnant and lactating women and children below the age of six years through anganwadi centres (AWCs), serving a catchment area of a population of 1,000 [28,29]. We selected the key informants based on their position, working experience, availability, and consent for the interview. Further, we selected 8-12 people from each block for Focus Group Discussion (FGD). The inclusion criteria for FGD included: any community member aged between 18-65 years who belong to the household where birth or death took place in the last five years and consented to participate in the study.

Data collection

KK and NS developed semi-structured questionnaires and selected the study area. KK conducted key informant interviews (n=15) and focus group discussions (8 FGD with 90 participants) with the local person's assistance to access the study area. KK went to block offices, primary and community health centres, Panchayat chief or Ward Parsad (local leader) office, and anganwadi centres, accessible to him. In addition, Assistant Registrar or BSO, Medical Officers and registrars at Nagar Nigam were invited for the interview. Fig 3 shows the hierarchy of CRS staff in Bihar. We also did telephonic interview with two CRS staff (Block Statistical Officers) to assess the operational status of the system during the pandemic.

Fig 3. Hierarchy of Civil Registration System in Bihar

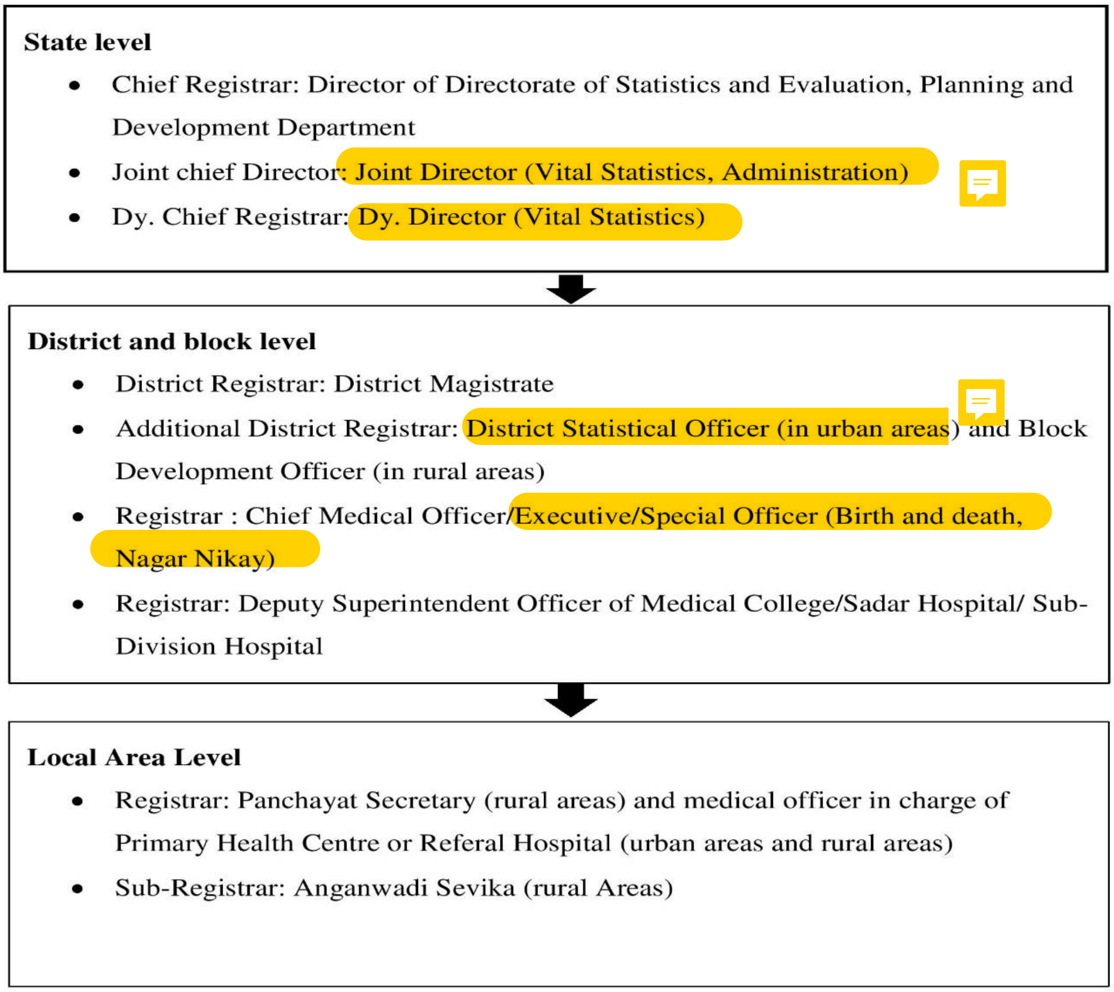

Source- CRS annual report, 2020. Department of Planning and Development, Bihar, 2021

Further, KK visited households with assistance from a Panchayat (a village council) Secretary or Ward Parsad and volunteer (postal worker, anganwadi sevika and teacher) of the area and invited an eligible household member (male or female) to participate in FGD. An eligible household member is a male or female adult of the household (18 years age and above) who can respond on behalf of the household. KK conducted 8 FGD, 1 FGD in each of the selected blocks, including 10-12 community members. KK noted all information during interviews with key informants to ensure no essential points were missed. In addition, KK took consent for note-taking and audio recording from all participants. A few community members (20 people) refused to participate, citing their reasons, and therefore, KK excluded them from participation. Each FGD consisted of a mix of male and female respondents. The demographic profile of all the study participants is shown in Table 1.

INSERT TABLE 1 HERE

Question schedules and topic guides were designed using previous literature [8, 23,30,31]. We used a semi-structured open-ended questionnaire to capture informants` experiences, stories, ideas, and case studies (see S1 Text). Questions were asked in the Hindi language. KK collected the data. KK also received seven days of training on the qualitative data collection method before the primary surveys. The confidentiality of participants fully adhered.

Reflexivity

At the time of conducting this study, the interviewer KK was an M.Phil. Student. The interviewer belonged to the Patna district of Bihar. KK first stressed his identity as belonging to the same state and speaking the same language (Hindi). KK built familiarity and rapport with the study participants. Local persons helped him in finding the eligible participants for the study. KK explained the study's objective to them, and gave assurance that the researcher had no personal ulterior motives for conducting the survey. KK did not pre-assume any results and entirely relied on the participants' spoken words.

Data Analysis

We anonymised all recorded notes and audio. We chose thematic analysis for obtaining a systematic framework to code qualitative data to identify patterns across the data [32]. The transcripts were transcribed in the local language (Hindi). Further, the recorded transcripts were translated into English to understand the theme by the wider population. KK coded transcripts and organised them by the sub-group of the participants. We used qualitative data software Atlas-ti 8.0 for the thematic analysis of data. We used direct quotes for exemplary purposes. The senior author (NS) reviewed typed transcripts for accuracy, completion, and plausibility. We also looked for the data saturation to validate our findings.

Ethics statement

We obtained informed consent from all the participants before any interviews and group discussions were conducted. The study protocols have been reviewed and approved by the institutional ethical review board at the Jawaharlal Nehru University (Ref. No. 2019/Student/235).

Results

Themes generated based on FGDs among community members

Based on the FGDs, we identified three main themes that were found to be critical barriers in the well-functioning of the system at the community level: 1) awareness and knowledge of registration procedures and the birth and death registration benefits, 2) people's attitude and perception towards birth and death registration and 3) administrative challenges experienced by the community members.

1. Awareness and knowledge of registration procedures and the birth and death registration benefits

Majority of FGDs' participants knew how to apply for birth and death registration. Among total FGDs' participants (n=90), nearly fifty percent registered their child's birth to anganwadi sevika and block officers. We found that a majority of study participants or their household female members delivered a baby in a public hospital. However, a subset of these participants (20 out of 60) reported their child's birth to anganwadi sevika, block officials, and Nagar Nigam offices due to no availability of dedicated staff and shortage of registration forms at the public health facilities. In addition, the requirement of identity cards was found to be barrier to birth

registration. According to the participants, identity proof (Aadhaar card, residential certificate, and ration card) is required for identity verification before birth and death registration. In the case of a child's birth registration, his or her parent's identity proof is required.

Aadhaar is a 12 digit individual identification number issued by the Unique Identification Authority of India on behalf of India's Government. This card serves as proof of identity and addresses anywhere in India. The district court issues a residential certificate about the place of birth and residence of the individuals. A ration card is also an official document issued by state governments in India to households eligible to purchase subsidised food grain from the Public Distribution System under the National Food Security Act. This card also serves as a common form of identification for Indian citizens.

"I received a birth certificate from the block office. My daughter's birth certificate was required for admitting her to school. Anganwadi sevika also demands my daughter's birth certificate for providing mid-day meal and benefits of government schemes such as financial assistance to the girl child." (25 years old female, Patna)

"I applied for my son's birth registration three months ago. My son got admission to the school without a birth certificate, so I did not go to the block for collecting his birth certificate. I also know a birth certificate is required to vaccinate him at a public health facility. I will go to the block office to collect the birth certificate when I get time." (32 years old man, Vaishali).

The registration law mandates all birth and death registration within 21 days. However, two-fifth of the total participants (n=90) applied for their child's birth registration after one year. A few participants mentioned that they applied for a child's birth registration when there was a requirement of a birth certificate for their child's enrolment in school. According to respondents, delayed registration is allowed on payment of five rupees (US $0.07) as delayed registration fees and an affidavit made before a notary public. A birth certificate is required for receiving benefits of government-aided services such as vaccination, financial support after a child's birth, and years of formal education. Birth registration was higher among female children because the Bihar government has initiated financial assistance scheme for girl children. We found that less than half of the study participants received birth certificates within one month. However, a subset of participants (10 out of 90) study participants did not receive a birth certificate for their child at all.

"A medical staff of a private hospital informed me that 2000 rupees would be deposited on my daughter's name by the government after her birth registration, after that, I reported my daughter's birth to a registrar at Nagar Nigam and requested a birth certificate." (25 years old female, Vaishali)

*"I applied for my daughter's birth registration after two years of her birth. I wanted to register her birth after her name ceremony. I paid ten rupees as delayed registration fees and requested the order of the District Magistrate for her birth registration." (*35 years old man, Patna*)*

Unlike birth registration, fewer participants (50 out of 90) report the death of their relatives or household members to civil authorities. We found that death registration was lower among females and infants. A few participants (15 out of 90) reported death at the registration centres situated at cemeteries or burial places. Besides, two-fifth of the study participants (36 out of 90) reported the death of their household members at the block or municipality offices. However, rural respondents said that there is no registration centre at the cemetery and burial place in their village; therefore, they are required to report to anganwadi sevika, panchayat secretary or block officials. A subset of participants (50 out of 90) said they submitted the deceased's Aadhaar card as identification proof. Most participants (75 out of 90) knew that a death certificate is mainly required to seek family pension, claim for insurance, and transfer property after a property holder's death.

"I reported the death of my uncle to the block office. A death certificate is required to seek a family pension, claim for insurance, and transfer property to another name after a property holder's death. Reporting death is also useful for medical officers to study the cause of death." (26 years old woman, Patna)

Nearly one-sixth of the total participants (15 out of 90) were unaware of the death registration procedures and benefits associated with death registration. A higher number of participants (35 out of 50) from Patna applied for birth and death registration of their household members than participants (19 out of 40) from Vaishali district. Rural and urban differentials in birth and death registration were found. Sometimes, people did not report births due to the lack of necessary documents or lack of support from the CRS officials.

"I did not know about the documents required for a child's birth registration. Also, I did not find anyone who could assist me in submitting the registration form at the block office; therefore, I did not report the birth of my daughter yet." (28 years old woman, Vaishali)

2. People's attitude and perceptions on birth and death registration

There was a high demand for birth certificates among community members. The majority of participants (70 out 90) reported birth registration of their household younger members because many immediate benefits are associated with birth registration. Nearly two-fifth of participants (36 out of 90) reported for their child's birth registration after a year due to traditional norms or local customs such as naming ceremonies. We found that participants gave more importance to the naming ceremony than the formal registration of the child's birth.

Most participants (50 out of 90) said that there was lower importance of death registration than birth registration. The death certificate was not required before cremation or burial. Some participants (40 out of 90) perceived death registration should be done within the prescribed time. A few participants (15 out of 90) also said it is not essential to report infant death whose birth was not registered. A few participants (10 out of 90)) did not report infant deaths because they believed that infant death is associated with sin of previous birth, and it should not be disclosed to others.

"In our tradition, a father cannot fire a dead body of his children. Death of an infant is considered a sin of parents' previous birth; therefore, people hide infant death from society members and hence, don't report". (38 years old man, Vaishali)

One important reason for not registering children's death was that there was no perceived necessity when the deceased did not possess property in their name. Also, there are no monetary or administrative incentives for registering deaths.

"I was aware that death is reported at the block or municipality office. However, I did not report my child's death because there were no benefits attached to it. Also, there was no property registered on his name." (35 years old man, Vaishali)

"I was aware that death registration is done at the block office and Nagar Nigam. However, I did not report the death of my wife to a civil registrar because a death certificate was not required as there was no property registered on her name." (40 years old man, Patna)

3. Administrative Challenges experienced by community members

About less than half of total respondents (40 out of 90) of FGD revealed that they faced problems in reporting their child's birth and household member's death. Some participants stood in a long queue for 3-4 hours at the blocks and Nagar Nigam offices. A few participants reported no water facility and shading for the people at the registration offices. Most of the participants (35 out of 90) took half day leave from their work to report their child's birth. Nearly 20 participants were asked to come the next day due to irregular electric current and no printing materials at the block offices. Besides, we found nearly two-fifths of the participants (35 out of 90) paid 50-200 rupees bribe to anganwadi sevika and block officers for receiving the birth or death certificate. However, a birth or death certificate should be provided free of cost by law. A few participants (10 out of 90) travelled 2-4 hours to reach registration offices and lost their wages for a day.

"*An officer told me there is no fixed time to provide the certificate. He also asked for 100 rupees for providing my son's birth certificate."* (23 years old man, Patna)

*"An anganwadi sevika registered my daughter's birth, but she demanded 100 rupees for providing the birth certificate. I did not have money at that time, so I did not collect the certificate. Later, I ignored her because I was not willing to give her 100 rupees for the birth certificate." (*30 years old man, Patna*)*

"I travelled 2 hours to reach the block office and stood 2-3 hours at the block office to report my father's death. I lost wage for a day and spent 100 rupees on travelling." (38 years old man, Vaishali)

We generated three themes based on KII with CRS staff : 1) Awareness and attitudes of the CRS staff, 2) Administrative and technical challenges, and 3) Staff's suggestions to improve birth and death registration level

1. Awareness and attitudes of the CRS staff

Among 15 staff interviewed, 12 staff showed good knowledge of laws and protocols associated with birth and death registration. They revealed birth and death certificates are provided free of cost, given registration was done within 21 days of the event. Moreover, delayed registration is allowed on submission of the affidavit, late fine and block and district level officers’ approval. We found most of the staff perceived timely and complete birth and death registration is crucial

for generating accurate vital statistics for policymaking. However, half of the total CRS staff interviewed did not read the CRS manual.

"I enter birth and death data into the system. A few times I feel difficult to assign correct codes in the birth and death registration forms due to unavailability of latest CRS manual in the office." (a CRS staff, Patna)

More than half of the total staff interviewed (10 out of 15) mentioned that targets were provided for birth and death registration by the office of Registrar General of India. The target for registration coverage is higher for births (>90%) than death registration (80%). These targets are the estimated number of birth and death to be registered by the CRS staff in a particular month. CRS manual shows the mathematical formula for calculating such a target. They also expressed that most of the medical attendant was not skilled in recording the cause of death.

"I received a monthly target of birth and death registration by the state headquarter. I don't know the mathematical formula for calculating the target. Also, I don't believe in such a target as institutional birth data are transferred directly to the Nagar Nigam and then district headquarter and not shared with me. Many times, the target is unreliable and difficult to achieve it". (a CRS staff, block office, Vaishali).

2. Administrative and technical challenges

Despite the government's announcement of the CRS portal for birth and death registration at a real-time basis in all the registration units, we found 1 out of 4 block offices of Patna, and 3 out of 4 block offices of Vaishali were not equipped with modern technologies such as computers, printers, photocopier and specific CRS software. Irregular electric supply and no separate department for CRS were observed.. We also found many staff of other department performed the duties of birth and death registration besides their departmental work.

"I am often given duty as an invigilator during the state's secondary board examination and other examination for screening of the candidates for public sector's jobs. I am also directed to assist officers in the conduct of municipalities or the state-level election." (a block-level officer, Patna)

This study found an anganwadi sevika performs multiple tasks such as providing pre-natal and post-natal care, assistance during birth delivery, and birth and death registration. They also teach children enrolled at their centre and serve mid-day meals to them. The pressure of performing multiple tasks limited their role as a sub-registrar and affected the quality of registration.

"Block officers assign multiple tasks to me. I am required to run an anganwadi centre where I teach and serve mid-day meals to the children . I also register birth or death occurs in this area." (an anganwadi sevika, Vaishali)

Furthermore, there was no regular training provided to the CRS staff. We found most of the CRS staff did not receive training in last one year. Anganwadi sevika was not invited in the block level meeting since last year. Besides, they were not provided with a birth and death registration forms and honorarium on time. In addition, there was also no adequate fund available for the periodic training and workshops for the staff.

"Block-level officers don't call me in the review meeting. I think monthly, or quarterly review meeting is required for solving problems encountered during birth and death registration." (an anganwadi sevika, Patna)

"I have not received any training in the last two years. I attended a training program on organising workshops on awareness on birth and death registration benefits two years back. I need training on how to operate CRS portal on a computer system." (a block-level officer, Patna)

There was no regular supervision at the block level or community level by the district or state level officers. Additionally, there was no strict implementation of the RBD Act, and the officers did not take any steps for improving the registration level in recent times, particularly death registration. We found health institution does delayed registration; however, it is not allowed as per official guidelines. We found poorer functioning of the registration system in the block offices of Vaishali as compared to the block offices of Patna.

3. Staff's suggestions to improve birth and death registration level

Most staff (10 out of 15) interviewed revealed that regular awareness programs on birth and death registration benefits could increase registration levels. Posters and banners exhibition during most celebrated festivals like Durgapuja, Chhat Puja, Ramnavmi procession, Harihar

Kshetra Mela (Sonepur cattle fair which attracts visitors from all over Asia) was widely suggested by the officials and staff. A few staff (5 out of 15) told mobile registration in hard-to-reach areas, or the villages far from registration centres could improve birth and death registration levels.

Further, medical personnel associated with CRS revealed that the birth and death reporting had increased after CRS' association with health facilities. Besides, the system manager, an assistant director, and block-level officers revealed that adequate awareness campaign on importance of death registration is needed to increase death reporting. In addition, they suggested that adequate funds should be provided for the smooth functioning of the system.

We also interviewed two CRS staff over telephone after third wave of Covid (January 2022) in India. They revealed that they did not achieve the birth and death registration level target due to shortage of staff. Majority of staff were appointed in Covid management duty. Therefore, the system failed to record birth and death on time during the pandemic.

Discussion

The present study investigated CRS's performance barriers in a poor state of India where birth and death registration is low. In 2012, there had been lower recording of birth and death. Again, there was slowdown in registration due to revamping of system in 2016 from paper-based registration to online system (CRS portal) for registration of vital events. Moreover, in order to accelerate the vital statistics quality and coverage and to create the awareness among the public, a descriptive analysis was published in the quarterly report of Directorate of Economics & Statistics namely “Bihar Sankhyaki Darpan”. The message on benefits of birth and death registration were also telecasted from Doordarshan Patna to attract the attention of rural and urban people towards registration [33].

The inadequate knowledge and attitude of community members on birth and death registration procedures and benefits are barriers to registration levels. Previous studies documented unawareness among people is a significant challenge in birth and death registration [2,23,31]. In our study area, knowledge on registration was not a barrier, yet we found a discouraging attitude, particularly to death registration, contributed to incomplete CRS in the study area. Perception

of little benefits of death reporting led to lower death registration in Bihar. The inadequate knowledge and perceptions of low benefits of birth and death registration limited the coverage of CRS in lower and middle-income countries [30,34].

Most people reported their child's birth to civil authorities because there are financial and social benefits associated with birth certificates. The government of Bihar encourages birth registration and provides financial assistance to female children after their birth registration [35]. Moreover, birth certificate is required for child’s school enrolment, vaccination and his social security [4,5]. Unlike reporting a child's birth, reasons for lower reporting for a person's death are not widely known. Consistent with previous studies, our study also documented a lower registration of infant and female deaths [24,25,27]. According to our participants, a lower death registration among females and children is due to a lack of legal necessity and no immediate financial or property-related benefits. We also observed that the presence of social stigma for premature death is negatively impacting death registration coverage. A lower reporting of infant deaths resulted from traditional beliefs such as an infant death as the sin of parent’s previous birth. For burial permission, reporting of death to civil authority was not required. Therefore, there is a lower priority for death registration among the participants. Underreporting of infant deaths that happen outside of facilities may lead to underestimating infant mortality.

In addition, differential reporting by sex will lead to insufficient quality data on sex differences in mortality rates [36]. Higher death registration of men could be associated with their engagement with the formal employment sector. Generally, family members are required to submit a death certificate to an employer to obtain social safety benefits after the death of a working member of the household. However, in our study areas, most of the respondents were engaged in the informal sector such as farming, construction of houses, a small business etc. that seldom provide social benefits to their families. Financial incentives for performing funeral activities and minimal financial assistance to deceased families may improve death reporting [37].

Even birth and death that occurred in health facilities was not recorded due to lack of dedicated staff and staff’s negligence. Public facilities record all birth and death but don't update on the CRS portal until birth and death certificates are requested by family members. A study

documented a similar argument for lower reporting of institutional birth and death in Bihar [38]. No strict implantation of the law is the primary reason for lower coverage of birth and death in Bihar. The previous study also revealed strict implementation and periodic audit of death registration is required [9,31,38,39].

This study highlighted poor services at the CRS offices such as a longer waiting time at the registration centres and not providing the certificates on time. In addition, the demand of bribes by CRS staff is discouraging for the participants not to report birth or death. Indirect opportunity costs such as travelling costs to the registration centre, loss of wage for a day were barriers to birth and death registration. Mobile registration in far-flung areas, reducing documentation work of applicants and assisting people in generating identity cards from concerned agencies would increase birth and death registration coverage. Moreover, awareness on specific cases like the adoption of a child and the death registration of a missing person should also be provided. anganwadi sevika, local leaders and district officials could be engaged in awareness programs with a minimal level of training to decrease negative impact of the customs or traditional beliefs on birth and death registration.

A knowledgeable and well-trained staff is essential for effective CRS [7]. This study showed staff had a good understanding of birth and death registration law and registration procedures. However, the lack of adequate training on the use of the CRS portal was a barrier to recording vital events. Department lack dedicated staff deputed solely for CRS work. Multiple tasks overburdened CRS officials led to compromise with the quality of vital statistics. The previous study also documented lack of dedicated staff, lack of training on the use of computer systems for recording birth and death was barriers to CRS functioning [9, 31]. Refresher training is also essential for updating and solving the fields' problems [7,38].

Regular monitoring of registration unit at primary and community healthcare, may ensure accessibility and improve birth registration level. Previous studies documented urbanisation and access to healthcare facilities are associated with higher birth registration [9,27,31,40]. An improvement of health facilities in Bihar is needed to improve coverage of birth and death registration. According to National Family Health Survey-2019-20, Bihar has one of the lowest maternal and child health care service utilisation within the country [41].

Telephonic interview with CRS staff reveal that during the Covid, the system failed to achieve the target of birth and death registration as there is shortage of CRS staff. Besides, staff were appointed on Covid management duty such as awareness program, monitoring and inspection of free supply of food, medicines and other healthcare items.

This study suggests some crucial points to improve birth and death registration levels. First, recurring awareness programs on birth and death registration benefits for CRS staff and community members particularly, rural residents. Customs or traditional beliefs led to lower reporting of vital event, should be changed with adequate awareness campaign. Second, there is a need to improve accessibility to registration centres and the use of ICT to speed up the CRS work. Third, adequate funds, dedicated staff and regular monitoring and auditing of the vital statistics may improve the registration level. Fourth, standardized operating protocol should be developed for uniform processes in the system because many registration offices are not aware of CRS manual and how to use CRS portal effectively. Fifth, state-wise registration target are set by Registrar General of India, however it is based on crude birth and death rate. We recommend state, district and sub-district level target needs to be worked out using some robust scientific methodology. The aspect of migration, live birth reported by health management information system should be taken into consideration while estimating registration target.

Despite a comprehensive analysis, this study has some limitations. This study covers only two districts of Bihar; therefore, generalisation could be made for the states or districts with a similar development level with care. We did not show comparison study with CRS operations in other states with similar level of development due to limited literature. We did not collected data by socio-economic background (such as education, wealth, religion or castes) of study participants; therefore, such analysis cannot be done in this study. In addition, this study is mainly focused on barriers at the operational level and not assessed the quality of vital statistics. Moreover, we did not check the birth and death certificate owned by respondents, so there could be under-reporting or over-reporting of birth and death registration due to recall bias. Possession of a birth certificate is considered socially desirable, so there might be bias reporting of birth registration.

Conclusions

Delayed or absent birth records could limit the government's ability to understand the population's needs and are associated with long-term health care use and outcomes. Lack of death registration and evidence of disparities in registration by sex could lead decision-makers target services inappropriately and lead demographers to make inaccurate estimations of trends. While support for those collecting this information is crucial, addressing socio-cultural beliefs and incentives for the death registration of certain people (infants, women) is also critical. Adequate awareness campaign is required to increase birth and death registration level in Bihar.

Acknowledgements

The authors would like to acknowledge study participants who co-operated and provided their valuable time for the in-depth interview and FGD. Besides, we are thankful to all the Ambedkar Library staff, Jawaharlal Nehru University, New Delhi, for providing access to Journals

Authors Contributions

Conceptualisation: Nandita Saikia (NS)

Data curation : Krishna Kumar (KK)

Formal analysis: Krishna Kumar

Methodology: Nandita Saikia & Krishna Kumar

Supervision: Nandita Saikia

Writing- Original draft: Krishna Kumar

Writing- review & editing: Nandita Saikia & Nadia Diamond-Smith

References

1. UNICEF. United Nations Children's Fund. Every Child's Birth Right: Inequities and trends in birth registration. UNICEF, New York. 2013. https://www.un.org/ruleoflaw/files/Embargoed_11_Dec_Birth_Registration_report_low_res.pdf Accessed on 2nd Oct 2021.
2. Office of Registrar General of India (ORGI). Vital Statistics of India based on the Civil Registration System 2019. New Delhi. 2021. https://censusindia.gov.in/2011-Common/CRS_2019/CRS2019_report.pdf Accessed on 25th Sep 2021.
3. UNICEF. Birth registration for every child by 2030: are we on track? UNICEF. 2019. https://reliefweb.int/sites/reliefweb.int/files/resources/Birth-registration-for-every-child-by-2030-brochure.pdf Accessed on 25th Nov 2020.
4. AbouZahr C, Cleland J, Coullare F, Macfarlane SB, Notzon FC, Setel P, Monitoring of Vital Events (MoVE) writing groups. The way forward. The Lancet. 2007; 370(9601): 1791-99. https://doi.org/10.1016/S0140-6736(07)61310-5
5. Duryea S, Olgiati A, Stone L. The under-registration of births in Latin America. 2006. IDB Working Paper No. 458. https://dx.doi.org/10.2139/ssrn.1820031
6. Setel PW, Macfarlane SB, Szreter S, Mikkelsen L, Jha P, Stout S, Monitoring of Vital Events (MoVE) writing groups. A scandal of invisibility: making everyone count by counting everyone. The Lancet. 2007; 370(9598):1569-77. https://doi.org/10.1016/S0140-6736(07)61307-5
7. United Nations. Principles and recommendations for a vital statistics system. United Nations. 2014. https://unstats.un.org/unsd/demographic/standmeth/principles/m19rev3en.pdf Accessed on 25 Sep 2021
8. WHO. Civil registration: why counting births and deaths is important. WHO. 2014 https://www.who.int/news-room/fact-sheets/detail/civil-registration-why-counting-births-and-deaths-is-important Accessed on 1st Mar 2021.
9. Oung MT, Richter K, Prasartkul P, Aung Y, Soe KT, Tin TC, Tangcharoensathien V. Reliable mortality statistics in Myanmar: a qualitative assessment of challenges in two townships. BMC Public Health. 2019;19(1):1-10. https://doi.org/10.1186/s12889-019-6671-y

10. Nojilana B, Groenewald P, Bradshaw D, Reagon G. Quality of cause of death certification at an academic hospital in Cape Town, South Africa. South African Medical Journal. 2009; 99(9). https://hdl.handle.net/10520/EJC69546
11. Rampatige R, Gamage S, Peiris S. Assessing the reliability of causes of death reported by the Vital Registration System in Sri Lanka: medical records review in Colombo. HIMJ. 2013; 42(3):20–28. https://doi.org/10.1177%2F183335831304200302
12. Australia Indonesia Partnership for Justice (AIPJ). Centre on Child Protection. Indonesia's missing millions. AIPJ baseline study on legal Identiy. 2014. http://www.cpcnetwork.org/wp-content/uploads/2015/02/AIPJ-PUSKAPA-BASELINE-STUDY-ON-LEGAL-IDENTITY-Indonesia-2013.pdf Accessed on 26 Mar 2021
13. UNICEF. The "Rights" Start to Life: A Statistical Analysis of Birth Registration. UNICEF. 2005. https://www.unicef.org/spanish/protection/rights_to_life.pdf
14. Zeng X, Adair T, Wang L, Yin P, Qi J, Liu Y, Liu J, Lopez AD, Zhou M. Measuring the completeness of death registration in 2844 Chinese counties in 2018. BMC medicine. 2020; 18(1):1-1. https://doi.org/10.1186/s12916-020-01632-8
15. Saikia N, Kulkarni PM. Data for Research into Health Inequality in India. The Economic and Political Weekly. 2016; 51(26):111-6.
16. Saikia N, Kulkarni PM. An assessment of India's readiness for tracking SDG Targets on health and nutrition. ORF Occasional Paper. 2018;108. https://www.orfonline.org/research/an-assessment-of-indias-readiness-for-tracking-sdg-targets-on-health-and-nutrition/
17. United Nations. Sustainable Development Goals: Goal 3. United Nations. https://www.un.org/sustainabledevelopment/health/ (accessed on 2 October 2021.)
18. International Institute for Population Sciences (IIPS) and ICF. National Family Health Survey (NFHS-4), 2015-16: India. Mumbai: IIPS. 2017. http://rchiips.org/NFHS/NFHS-4Report.shtml
19. Office of Registrar General of India (ORGI). Vital Statistics of India based on the Civil Registration System 2014. New Delhi. 2016. https://www.censusindia.gov.in/2011-Documents/CRS_Report/crs2014_final.pdf Accessed on 21 Sep 2020.
20. Office of Registrar General of India (ORGI). Vital Statistics of India based on the Civil Registration System 2017. New Delhi. 2019. http://crsorgi.gov.in/web/uploads/download/CRS_report_2017_20192020.pdf Accessed on 29 Sep 2020.

21. Office of Registrar General of India (ORGI). Vital Statistics of India based on the Civil Registration System 2018. New Delhi. 2020. https://censusindia.gov.in/2011-Common/CRS_2018/crs2018_20072020.pdf Accessed on 2 October 2021.
22. Hill K, Lopez AD, Shibuya K. Interim measures for meeting needs for health sector data: births, deaths, and causes of death. The Lancet. 2007;370(9600):1726-35. https://doi.org/10.1016/S0140-6736(07)61309-9
23. Li S, Zhang Y, Feldman MW. Birth registration in China: practices, problems and policies. Population Research and Policy Review. 2010;29(3):297-317. https://doi.org/10.1007/s11113-009-9141-x
24. Gupta M, Rao C, Lakshmi PV, Prinja S, Kumar R. Estimating mortality using data from civil registration: a cross-sectional study in India. Bulletin of the World Health Organization. 2016;94(1):10. https://dx.doi.org/10.2471%2FBLT.15.153585
25. Kumar, G. A., Dandona, L., & Dandona, R. (2019). Completeness of death registration in the Civil Registration System, India (2005 to 2015). *The Indian journal of medical research*, *149*(6),740-47. https://dx.doi.org/10.4103%2Fijmr.IJMR_1620_17
26. Rao C, Gupta M. The civil registration system is a potentially viable data source for reliable subnational mortality measurement in India. BMJ Global Health 2020;5:e002586. http://dx.doi.org/10.1136/bmjgh-2020-002586
27. James K S, Kavita N, George A. Preliminary assessment of the quality of civil registration system in Kerala, Odisha and Rajasthan. UNFPA. 2013. http://india.unfpa.org/sites/default/files/pub-pdf/ReportonCRS-Final.pdf
28. Rao N, Kaul V. India's integrated child development services scheme: challenges for scaling up. Child Care Health Dev. 2018; 44(1):31–40. https://doi.org/10.1111/cch.12531 PMID: 29235171
29. Gopalakrishnan L, Buback L, Fernald L, Walker D, Diamond-Smith N, in addition to The CAS Evaluation Consortium. Using mHealth to improve health care delivery in India: A qualitative examination of the perspectives of community health workers and beneficiaries. PLoS ONE. 2020; 15(1): e0227451. https://doi.org/10.1371/journal.pone.0227451
30. Haider MM, Alam N, Ibn Bashar M, Helleringer S. Adult death registration in Matlab, rural Bangladesh: completeness, correlates, and obstacles. Genus. 2021; 77 https://doi.org/10.1186/s41118-021-00125-7

31. Rane TM, Mahanta TG, Islam S, Gogoi PP, Gogoi B. Civil registration system (CRS) for birth and death registration in Assam–a rapid assessment. Clinical Epidemiology and Global Health. 2020; 8(1):117-22. https://doi.org/10.1016/j.cegh.2019.05.006
32. Braun V, Clarke V. Using thematic analysis in psychology. Qualitative research in psychology. 2006;3(2):77-101.
33. Office of Registrar General of India (ORGI). Vital Statistics of India based on the Civil Registration System 2012. New Delhi. 2015. https://censusindia.gov.in/2011-Documents/CRS_Report/CRS_Report2012.pdf

34. Fisker A, Rodrigues A, Helleringer S. Diferences in barriers to birth and death registration in Guinea-Bissau: Implications for monitoring national and global health objectives. Tropical Medicine and International Health. 2019;24(2),166–174. https://doi.org/10.1111/tmi.13177
35. Women Development Corporation. Mukhyamantri Kanya Suraksha Yojna. Bihar. https://wdc.bih.nic.in/MKSYDetails.aspx Accessed on 2 October 2021.
36. Adair T, Gamage USH, Mikkelsen L, et al. Are there sex differences in completeness of death registration and quality of cause of death statistics? Results from a global analysis. BMJ Global Health 2021;6:e006660. http://dx.doi.org/10.1136/bmjgh-2021-006660
37. Garenne M, Collinson MA, Kabudula CW, Gómez-Olivé, FX, Kahn K, Tollman S. Completeness of birth and death registration in a rural area of South Africa: The Agincourt health and demographic surveillance, 1992–2014. Global Health Action. 2016; 9(1), 32795. https://doi.org/10.3402/gha.v9.32795.
38. Kumar D, Pandey BK, Kumar Ajit, Basu JK. Civil Registration in Bihar: Strengths and Challenges. Demography India. 2021.
39. Patel AB, Rathod H, Rana H, Patel V. Assessment of Medical certificate of medical certificate of cause of death at a new teaching hospital in Vadodara. Natl J Commun Med. 2011;2(3):349–353.
40. Fall A, Masquelier B, Niang K., Ndiaye S, Ndonky A. Motivations and barriers to death registration in Dakar, Senegal. *Genus*.2021;*77*(1), 1-20. https://doi.org/10.1186/s41118-021-00133-7
41. Ministry of Health and Family Welfare & International Institute for Population Sciences (IIPS). Bihar Fact Sheet. National Family Health Survey (NFHS-5) 2019-20. India. Mumbai: IIPS. 2021. http://rchiips.org/nfhs/NFHS-5_FCTS/FactSheet_BR.pdf

Supporting information

S1 Text. Guide for KII and FGD.

S1 Table. Rate (%) of health and education indicators in the Bihar and India, 2015-2016.

S2 Table. Names of blocks of Patna and Vaishali.

S3 Table. Estimates of birth and death registration level by districts of Bihar

S3 Text 3. COREQ checklist.

Table 1. Demographic profile of the study participants

	Mean age (in years) & (range)	Study Area				Number of respondents	Method of data collection
		Patna		Vaishali			
		Male	Female	Male	Female		
**Assistant Director**	50	1	-	-	-	1	KII
**System Manager**	35	1	-	-	-	1	KII
**BSO**	41 (40-42)	1	-	1	-	2	KII
**Anganwadi Sevika**	44.3 (40-50)	-	2	-	2	4	KII
**Medical officer**	52.2 (45-62)	-	2	1	-	3	KII
**Ward Parsad**	29.5 (24-35)	1	-	1	-	2	KII
**Nagar Nigam**	36 (30-42)	1	-	1	-	2	KII
**Community members**	35.6 (17-62)	30	13	25	22	90	FGD

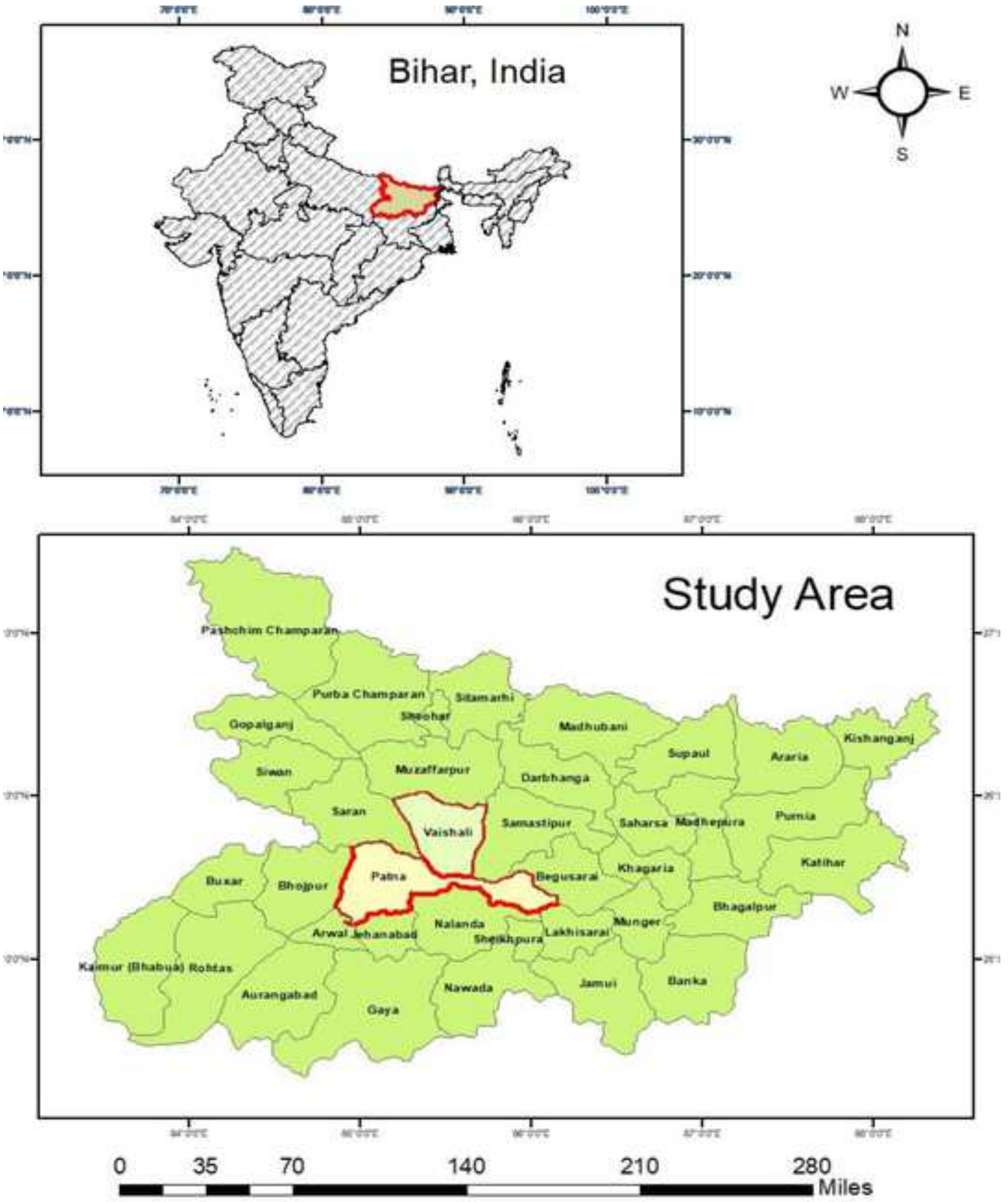
Bihar, India
Study Area
N
W
E
S
Pashchim Champaran
Purba Champaran
Sitamarhi
Sheohar
Gopalganj
Madhubani
Kishanganj
Supaul
Araria
Siwan
Muzaffarpur
Darbhanga
Saran
Vaishali
Samastipur
Saharsa
Madhepura
Purnia
Katihar
Khagaria
Buxar
Bhojpur
Patna
Begusarai
Bhagalpur
Munger
Arwal
Jehanabad
Nalanda
Sheikhpura
Lakhisarai
Kaimur (Bhabua)
Rohtas
Jamui
Banka
Aurangabad
Gaya
Nawada
0 35 70 140 210 280
Miles

Click here to access/download
Supporting Information
S1 Table.docx

Click here to access/download
Supporting Information
S2 Table.xlsx

Click here to access/download
Supporting Information
S3 Table.xls

Click here to access/download
Supporting Information
S3 Text. COREQ checklist.doc

Revised Manuscript with Track Changes

Performance barriers of Civil Registration System in Bihar: An exploratory study

Krishna Kumar[1], Nandita Saikia[2*], Nadia Diamond-smith[3]

[1]Centre for the Study of Regional Development, School of Social Sciences III, Jawaharlal Nehru University, New Delhi, India 110067

Email: krishhna94@gmail.com

ORCID iD: http://orcid.org/0000-0002-6822-900X

[2][2]~~Centre for the Study of Regional Development, School of Social Sciences III, Jawaharlal Nehru University, New Delhi, India 1~~Department of Public Health and Mortality Studies, International Institute for Population Sciences, Deonar, Mumbai-400088~~10067~~

Email: nanditasts@gmail.com

ORCID iD: https://orcid.org/0000-0001-6735-6157

[3]Epidemiology and Biostatistics Department, University of California, San Francisco CA 94158

Email: Nadia.Diamond-Smith@ucsf.edu

ORCID iD: https://orcid.org/0000-0002-8711-3029

***Corresponding author:**

Nandita Saikia

Email: nanditasts@gmail.com

Running Title: Civil Registration System in Bihar

Word Count: 6636~~6264~~

Abstract

Objectives: Vital statistics generated by the Civil Registration System (CRS) are essential for developing healthcare interventions at all administrative levels. Among all India's states, Bihar showed a lower recording of birth and death in 2019. This study investigates CRS's performance barriers from the perspective of CRS staff and community members in Bihar.

Methods: We conducted a primary qualitative survey in the two districts of Bihar during February-March 2020 with CRS staff (n=15) and community members (n=90). We purposively selected the Patna and Vaishali districts of Bihar for the survey. Thematic analysis was done to identify the pattern across the data using the Atlas-ti software.

Results: Most participants showed a good understanding of registration procedures and birth and death registration benefits. The perceived need for death registration is lower than birth registration. While birth registration was higher among female children than male children, death registration was lower among females. We found that most participants did not report children or adult female death due to lack of financial or property-related benefits. Death registration was not mandatory for burial or cremation permits. Most participants faced challenges in reporting birth and death due to poor delivery of services at the registration centres, higher indirect opportunity cost, and demand of bribes by the CRS staff for providing certificates. We found a lack of adequate investment, a dedicated staff shortage, and limited computer and internet services at the registration ~~centres~~centers. ~~Additionally, there was no strict implementation of the birth and death registration law in the study area.~~

Conclusions: ~~Lack of~~ Poor data on birth and death registration could lead decision-makers ~~not~~ to target health services inappropriately. Strengthening health institutions' linkage with the registration centres, mobile registration in far-flung areas and regular CRS staff training could increase death registration levels. ~~Besides, mandatory burial or cremation permits may increase the demand for death certificates.~~~~Adequate~~An adequate awareness campaign ~~-~~on the benefits of birth and death registration is required to increase the reporting of vital events.

Introduction

A Civil Registration System (CRS) is a permanent, continuous, compulsory, and universal recording of vital events with the legal requirements [1]. The Civil Registration Vital Statistics ~~(CRVS)~~ is exact and actual data, certified by registering authority, and legally acceptable [2]. A birth certificate documents information such as age, place of birth, and family background [3]. Besides official documentation of a child's birth, it facilitates government-aided essential services such as education, health facilities, and social security [4-6]. CRS also provides timely and reliable vital statistics. Health officials and policymakers mainly use CRS data to track fertility, mortality, and epidemiological patterns at all administrative levels. United Nations documented that a functional CRS is the best source of information on vital events for administrative, demographic, and epidemiological purposes [7].

Despite government agencies and UNICEF's effort to ~~universalise~~ universalize birth registration globally, about 166 million children under age five and 40 million infants were not officially documented [3]. According to UNICEF's report (2019), about 77 million children under age five do not have a birth certificate in South Asia. There has been a significant disparity among countries in terms of birth registration. Low or middle-income countries showed more unsatisfactory performance in registering the child's birth. Mortality statistics are also essential for identifying health risks and evaluating health programs. In many countries, the completeness of death registration is lower than birth registration. World Health Organisation (WHO) recorded about the ~~two-third~~two-thirds of global deaths were not registered. Besides, most WHO members obtained incomplete data on mortality and the cause of death [8]. Another study showed that administrative challenges, insufficient technical capacities, and unawareness are significant challenges in death registration [9]. Accurate recording of death's cause is challenging in lower and middle-income countries [10,11].

In India, the Births, Deaths, and Marriage Registration (RBD) Act, enacted in 1886, suggested voluntary registration of births and deaths. However, it was not uniformly implemented across India. After independence, India's Government introduced the Registration of Births and Deaths Act in 1969, which mandates registration of all births and deaths within 21 days [2]. A low ~~Lack~~

~~of~~ birth registration is associated with increased school dropout, child trafficking, and child labor [1,12]. A higher~~The~~ child mortality rate is associated with higher level of ~~higher among~~ unregistered children [13]. A lower death registration limits medical research and reliable mortality estimates [2,14]. Despite India's mandatory birth and death registration law, according to official statistics, nearly 7 percent of births and 8 percent of deaths were not registered in India in 2019 [2]. Further, there are disparities at the state and district level regarding coverage and completeness of birth and death registration. In India, seventy percent of the districts have more than a million population each; lower death registration coverage at the district level precludes estimating health indicators with precision [15]. Universal civil registration in the interest of sound policymaking and its implementation helps monitor the SDGs goals such as promoting healthy lives and well-being, reducing mortality rate and legal identity for all [16, 17].

Bihar is one of the least developed and third most populated state~~s~~ of India. A comparison table of demographic and health indicators of Bihar and India is shown in S1 Table. The total population of Bihar is 113 million (which is equivalent to the population size of Ethiopia, the world's 12th populous country), constituting 8.57% of India's population. According to the National Family Health Survey, 2015-16, Bihar has the highest total fertility rate (3.14) in India. The infant death rate and under-five mortality in Bihar are 48.1 and 58.1 per thousand live births. Education attainment is quite low, with only 14 percent of the household population of eighteen and above completed 12 or more years of schooling. Among the children, age 0-6 years, 41 percent and 33 percent received immunisation and health check-up from an anganwadi sevika. The percentage of institutional birth is lower in Bihar (64%) as compared with ~~the~~ India level (79%) [18]. In Bihar, birth and death registration levels are much lower than the national average. However, there has been an increase in birth registration levels in Bihar from 64% in 2014 to 89% in 2019 [2,19]. On the other hand, the death registration level was lower (52%) in Bihar compared with other Indian states in 2019 [2]. Additionally, Bihar showed inconsistency in death registration level from 43% in 2017 to 35% in 2018 and further, the level increased to 52 percent in 2019 [2,20,21].

There has been increased research around the impact of under-registration, but existing studies focused on the need and benefits associated with functional registration systems [6,22,23]. Recent studies primarily focused on birth and death registration levels, trends and estimation of

death registration level using indirect methods [(24,25,26]). However, such studies did not answer why death registration is lower in some states of India. While mMedia reports and public health experts have highlighted the importance of up-to-date vital statistics for initiating healthcare intervention during a health emergency, peer-reviewed literature investigating motivations and barriers to birth and death registration in India is limited. Previous studies also suggested a primary qualitative study studies in the states or districts where a lower registration level was recorded [7,9,27] to explore the contextual determinants of under registration [7,9,27]. As of our knowledge, peer-reviewed literature investigating motivations and barriers to birth and death registration is limited and not widely discussed. This study investigates the performance barriers of the CRS in Bihar, a state with the lower level- of birth and death registration. This study mayprovided crucial inputs to -contributes to knowledge of reform administrative reforms issues -for an effective CRS in Bihar.

Materials and Methods

We conducted a primary qualitative survey in the two districts of Bihar during February-March 2020 with CRS staff (n=15) and community members (n=90). Fig 1 shows the framework of the operation of CRS in Bihar.

Formatted: Font: 11 pt, Bold

Formatted: Font: 11 pt

Fig 1. Flow chart of birth and death registration in Bihar

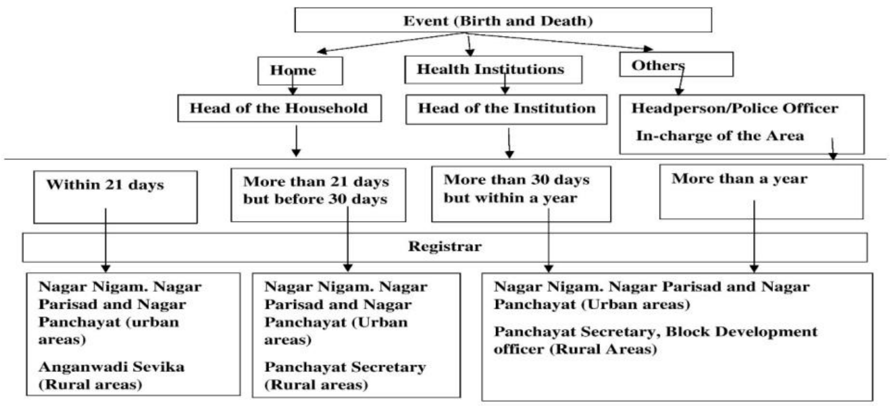

CRS annual report, 2020. Department of Planning and Development, Bihar, 2021

Note:- In case of birth and death registration after 30 days but within 1 year, registration is done after written permission of the prescribed authority and on the submission of an affidavit made before a notary public and payment of a late fee of rupee five (US$ 0.07)

In case of registration of birth and death after an year, an order is required by a magistrate of the first class (Block Development Officer) after verifying the correctness of the event and payment a fee of rupee ten (US$ 0.14)

~~S~~Study design and sample

Fifteen CRS staff and ninety community members participated in the survey. Out of 38 districts in Bihar, we purposively chose two districts Patna and Vaishali, from which we randomly selected four blocks from each district. Fig 2 shows the study area. We chose Patna and Vaishali districts to investigate how registration practices differ in two different ~~context~~contexts. These districts represent two different urbaniz~~s~~ation contexts and registration levels: Patna being the most ~~urbanised~~ urbanized (43%) and Vaishali the least urbanised district (7%) of Bihar. Patna has better health care facilities, a better road network, CRS offices equipped with modern technologies, death registration level (61%) and a higher literate population (71%). On the other hand, Vaishali has a poor road network and transport facilities, lower access to public and private health institutions, death registration level (45%) and a lower proportion of the literate population (67%) See S3 table (sheet 3). We selected Patna Sadar, Sampatchak, Phulwarisarif,

Formatted: Font: 9 pt, Bold
Formatted: Font: 12 pt, Bold
Formatted: Font: 12 pt
Commented [D1]: Giving arrow sign in the boxes will look better
Formatted: Justified
Formatted: Not Highlight
Formatted: Not Highlight
Formatted: Not Highlight
Formatted: Not Highlight
Formatted: Not Highlight
Formatted: Not Highlight
Formatted: Not Highlight
Formatted: Not Highlight
Formatted: Not Highlight

and Danapur blocks from Patna, and Hajipur, Vaishali, Desri, and Raghopur blocks from Vaishali. 4 out of total 22 blocks of Patna were randomly selected using a RANDBETWEEN function in excel. Similarly, 4 out of 16 blocks of Vaishali were selected (see~~-~~ S2 Table). We also showed estimated birth and death registration level by districts of Bihar (See S3 Table).

INSERT Fig 2 HERE

Source- Authors generated the map using GIS

Study participants

Block Statistical Officer (BSO), a Medical Officer, a Panchayat Chief or Ward Parsad (local leader), ~~and~~ an Anganwadi Sevika and a registrar at Nagar Nigam ~~-~~from each block were chosen for the key informant interview (KII). An a~~A~~nganwadi s~~S~~evika is a community health worker from the local village who delivers services to pregnant and lactating women and children below the age of six years through a~~A~~n~~n~~ganwadi c~~C~~entres (AWCs), serving a catchment area of a population of 1,000 [28,29]. We selected the key informants based on their position, working experience, availability, and consent for the interview. Further, we selected 8-12 people from each block for Focus Group Discussion (FGD). The inclusion criteria for FGD included: any community member aged between 18-65 years who belong to the household where birth or death took place in the last five years and consented to participate in the study.

Data collection

KK and NS developed semi-structured questionnaires and selected the study area. KK conducted key informant interviews (n=15) and focus group discussions (8 FGD with 90 participants) with the local person's assistance to access the study area. KK went to block offices, primary and community health centres, Panchayat chief or Ward Parsad (local leader) office, and a~~A~~nganwadi centres, accessible to him. In addition, Assistant Registrar or BSO, Medical Officers and registrars at Nagar Nigam ~~-~~were invited for the interview. Fig 3 shows the hierarchy of CRS staff in Bihar. We also did telephonic interview with two CRS staff (Block Statistical Officers) to assess the operational status of the system during the pandemic.

Fig 3. Hierarchy of Civil Registration System in Bihar

Formatted: Space Before: 0 pt, Tab stops: Not at

Formatted: Space Before: 0 pt, Tab stops: Not at

State level

- Chief Registrar:
  (Director of sof

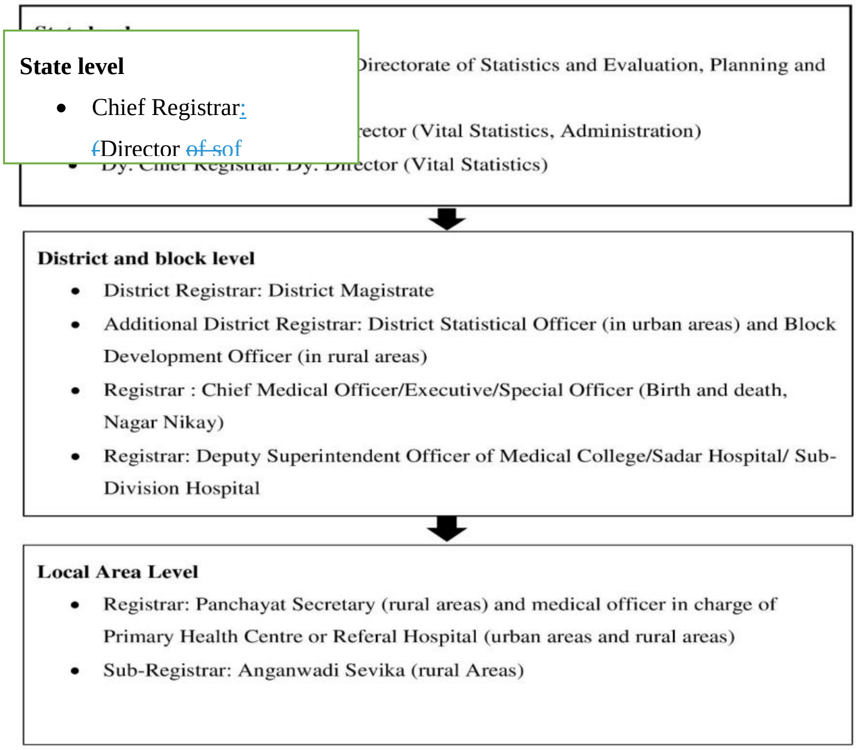

~~Figure 3. Hierarchy of Civil Registration System in Bihar~~

Formatted: Indent: First line: 0", Tab stops: 5.77", Left

Formatted: Tab stops: 5.77", Left

District and block level

- District Registrar: District Magistrate
- Additional District Registrar: District Statistical Officer (in urban areas) and Block Development Officer (in rural areas)
- Registrar : Chief Medical Officer/Executive/Special Officer (Birth and death, Nagar Nikay)
- Registrar: Deputy Superintendent Officer of Medical College/Sadar Hospital/ Sub-Division Hospital

Local Area Level

- Registrar: Panchayat Secretary~~vak~~ (rural areas) and medical officer in charge of Primary Health Centre or Referal Hospital (urban areas and rural areas)

  ~~& Medical officer of civil and district hospitals~~
- Sub-Registrar: Anganwadi Sevika (rural Areas)

Source- CRS annual report, 2020. Department of Planning and Development, Bihar, 2021

*

~~Figure 3 is obtained from ORGI report, 2021.~~

Further, KK visited households with assistance from a Panchayat (a village council) Secretary~~Chief~~ or Ward Parsad and volunteer (postal worker, anganwadi sevika and teacher) of the area and invited an eligible household member (male or female) to participate in FGD. An eligible household member is a male or female adult of the household (18 years age and above) who can respond on behalf of the household. KK conducted 8 FGD, 1 FGD in each of the selected blocks, including 10-12 community members. KK noted all information during interviews with key informants to ensure no essential points were missed. In addition, KK took consent for note-taking and audio recording from all participants. A few community members (20 people) refused to participate, citing their reasons, and therefore, KK excluded them from participation. Each FGD consisted of a mix of male and female respondents. The demographic profile of all the study participants is shown in Table 1.

INSERT TABLE 1 HERE

Question schedules and topic guides were designed using previous literature [8, 23,30,31]. We used a semi-structured open-ended questionnaire to capture informants` experiences, stories, ideas, and case studies (see S1~~1~~ Text). Questions were asked in the Hindi language. KK collected

Formatted: Font: 12 pt

Formatted: Bulleted + Level: 1 + Aligned at: 0.25" + Indent at: 0.5"

Formatted: Font: Not Bold

Formatted: Space Before: 6 pt, Tab stops: 5.77", Left

the data. KK also received seven days of training on the qualitative data collection method before the primary surveys. The confidentiality of participants fully adhered.

Reflexivity

At the time of conducting this study, the interviewer KK was an M.Phil. Student. The interviewer belonged to the Patna district of Bihar. KK first stressed his identity as belonging to the same state and speaking the same language (Hindi). KK built familiarity and rapport with the study participants. Local persons helped him in finding the eligible participants for the study. KK explained the study's objective to them, and gave assurance that the researcher had no personal ulterior motives for conducting the survey. KK did not pre-assume any results and entirely relied on the participants' spoken words.

Data Analysis

We anonymised all recorded notes and audio. We chose thematic analysis for obtaining a systematic framework to code qualitative data to identify patterns across the data [32]. The transcripts were transcribed in the local language (Hindi). Further, the recorded transcripts were translated into English to understand the theme by the wider population. KK coded transcripts and organised them by the sub-group of the participants. We used qualitative data software Atlas-ti 8.0 for the thematic analysis of data. We used direct quotes for exemplary purposes. The senior author (NS) reviewed typed transcripts for accuracy, completion, and plausibility. We also looked for the data saturation to validate our findings.

Ethics statement

We obtained informed consent from all the participants before any interviews and group discussions were conducted. The study protocols have been reviewed and approved by the institutional ethical review board at the Jawaharlal Nehru University (Ref. No. 2019/Student/235).

Results

Themes generated based on FGDs among community members

Based on the FGDs, we identified three main themes that were found to be critical barriers in well ~~–~~functioning of the system at the community level: 1) awareness and knowledge of registration procedures and the birth and death registration benefits, 2) people's attitude and perception towards birth and death registration and 3) administrative challenges experienced by the community members.

1. Awareness and knowledge of registration procedures and the birth and death registration benefits

Majority of ~~ost participants of the~~ FGDs' participants knew how to apply for birth and death registration. Among ~~Almost half of the~~ total FGDs' participants (n=90), nearly fifty percent registered their child's birth to a~~A~~nganwadi s~~S~~evika and block officers. We found that ~~although~~ a majority of study participants or their household female members delivered a baby in a public hospital. However, a subset ~~more than half~~ of these ~~participants~~ participants (20 out of 60) reported their child's birth to a~~A~~nganwadi s~~S~~evika, ~~or~~ block officials and Nagar Nigam offices due to no availability of dedicated staff and shortage of registration forms at the public health facilities. In addition, requirement~~s~~ of identity cards ~~and complex registration form~~ was found to be barriers to birth registration. According to the participants, identity proof (Aadhaar card, residential certificate, and ration card) is required for identity verification before birth and death registration. In the case of a child's birth registration, his or her parent's identity proof is required.

Aadhaar is a 12 digit individual identification number issued by the Unique Identification Authority of India on behalf of India's Government. This card serves as proof of identity and addresses anywhere in India. The district court issues a residential certificate about the place of birth and residence of the individuals. A ration card is also an official document issued by state governments in India to households eligible to purchase subsidised food grain from the Public Distribution System under the National Food Security Act. This card also serves as a common form of identification for Indian citizens.

"I received a birth certificate from the block office. My daughter's birth certificate was required for admitting her to school. Anganwadi s~~S~~evika also demands my daughter's birth certificate for providing ~~vaccination~~ mid-day meal and benefits of government schemes such as financial assistance to the girl child." (25 years old female, Patna)

"I applied for my son's birth registration three months ago. My son got admission to the school without a birth certificate, so I did not go to the block for collecting his birth certificate. I also know a birth certificate is required to vaccinate him at a public health facility. I will go to the block office to collect the birth certificate when I get time." (32 years old man, Vaishali).

The registration law mandates all birth and death registration within 21 days. However, two-fifth of the total participants (n=90) applied for their child's birth registration after one year. A few participants mentioned that they applied for a child's birth registration when there was a requirement of a birth certificate for their child's enrolment in school. According to respondents, delayed registration is allowed on payment of five~~10~~ rupees (US $0.07~~14~~) as delayed registration fees and an affidavit made before a notary public. A birth certificate is required for receiving benefits of government-aided services such as vaccination, financial support after a child's birth, and years of formal education. Birth registration was higher among female children because the Bihar government has initiated financial assistance scheme for girl children~~ to encourage birth registration~~. We found that less than half of the study participants received birth certificates within one month. However, a subset of participants (~~n=~~10~~)~~ out of 90) study participants did not receive a birth certificate for their child at all.

"*A medical staff of a private hospital informed me that 2000 rupees would be deposited on my daughter's name by the government after her birth registration, after that, I reported my daughter's birth to a registrar at Nagar Nigam and requested a birth certificate."* (25 years old female, Vaishali)

*"I applied for my daughter's birth registration after two years of her birth. I wanted to register her birth after her name ceremony. I paid ten~~10~~ rupees as delayed registration fees and requested the order of the District Magistrate for her birth registration." (*35 years old man, Patna*)*

Unlike birth registration, fewer participants (50 out of 90) report the death of their relatives or household members to civil authorities. We found that death registration was lower among females and infants. A few participants (~~n=~~15 out of 90) reported death at the registration centres situated at cemeteries or burial places. Besides, two~~two~~-fifth of the study participants (36 out of 90) reported the death of their household members at the block or municipality offices. However,

rural respondents said that there is no registration centre at the cemetery and burial place in their village; therefore, they are required to report to a~~A~~nganwadi sevika, panchayat secretary~~i~~ or block officials. A subset of participants (50 out of 90) said they submitted the deceased's Aadhaar card as identification proof. Most participants (75 out of 90) knew that a death certificate is mainly required to seek family pension, claim for insurance, and transfer property after a property holder's death.

"I reported the death of my uncle to the block office. A death certificate is required to seek a family pension, claim for insurance, and transfer property to another name after a property holder's death. Reporting death is also useful for medical officers to study the cause of death." (26 years old woman, Patna)

Nearly one-sixth of the total participants (15 out of 90) were unaware of the death registration procedures and benefits associated with death registration. A higher number of participants (35 out of 50) from Patna applied for birth and death registration of their household members than participants (19 out of 40) from Vaishali district. Rural and urban differentials in birth and death registration were found. Sometimes, people did not report births due to the lack of necessary documents or lack of support from the CRS officials.

"I did not know about the documents required for a child's birth registration. Also, I did not find anyone who could assist me in submitting the registration form at the block office; therefore, I did not report the birth of my daughter yet." (28 years old woman, Vaishali)

2. People's attitude and perceptions on birth and death registration

There was a high demand for birth certificates among community members. The majority of participants (70 out 90) reported birth registration of their household younger members because many immediate benefits are associated with birth registration. Nearly two-fifth of participants (36 out of 90) reported for their child's birth registration after a year due to traditional norms or local customs such as naming ceremonies. We found that participants gave more importance to the naming ceremony than the formal registration of the child's birth.

Most participants (50 out of 90) said that there was lower importance of death registration than birth registration. The death certificate was not required before cremation or burial. Some

participants (40 out of 90) perceived death registration should be done within the prescribed time. A few participants (15 out of 90) also ~~believed~~ said it is not essential to report infant death whose birth was not registered. A few participants (10 out of 90)~~(n=8~~) did not report infant deaths because they believed that infant death is associated with sin of previous birth, and it should not be disclosed to others.

"In our tradition, a father cannot fire a dead body of his children. Death of an infant is considered a sin of parents' previous birth; therefore, people hide infant death from society members and hence, don't report~~.~~". (38 years old man, Vaishali)

One important reason for not registering children's death was that there was no perceived necessity when the deceased did not possess property in their name. Also, there are no monetary or administrative incentives for registering deaths.

"I was aware that death is reported at the block or municipality office. However, I did not report my child's death because there were no benefits attached to it. Also, there was no property registered on his name." (35 years old man, Vaishali)

"I was aware that death registration is done at the block office and ~~municipal corporation.~~Nagar Nigam. However, I did not report the death of my ~~deceased~~ wife to a civil registrar because a death certificate was not required as there was no property registered on her name." (40 years old man, Patna)

3. Administrative Challenges experienced by community members

About less than half of total respondents (40 out of 90) of FGD revealed that they faced problems in reporting their child's birth and household member's death. Some participants stood in a long queue for 3-4 hours at the blocks and ~~municipal~~ Nagar Nigam offices. A few participants reported no water facility and shading for the people at the registration offices. Most of the participants (35 out of 90~~n=35~~) took half day leave from their work to report their child's birth. Nearly 20 participants were asked to come the next day due to irregular electric current and no printing materials at the block offices. Besides, we found nearly two~~hree~~-fifths of the participants (35 out of 90) paid 50-200 rupees bribe to an~~A~~ganwadi s~~S~~evika and block officers for receiving the birth or death certificate. However, a birth or death certificate should be provided free of cost by law. A few participants (10 out of 90) travelled 2-4 hours to reach registration offices and lost their wages for a day.

"An officer told me there is no fixed time to provide the certificate. He also asked for 100 rupees for providing my son's birth certificate." (23 years old man, Patna)

*"An a~~a~~nganwadi s~~s~~evika registered my daughter's birth, but she demanded 100 rupees for providing the birth certificate. I did not have money at that time, so I did not collect the certificate. Later, I ignored her because I was not willing to give her 100 rupees for the birth certificate." (*30 years old man, Patna*)*

"I travelled 2 hours to reach the block office and stood 2-3 hours at the block office to report my father's death. I lost wage for a day and spent 100 rupees on travelling." (38 years old man, Vaishali)

We generated three themes based on KII with CRS staff : 1) Awareness and attitudes of the CRS staff, 2) Administrative and technical challenges, and 3) Staff's suggestions to improve birth and death registration level

1. Awareness and attitudes of the CRS staff

Among 15 staff interviewed, 12 staff showed good knowledge of laws and protocols associated with birth and death registration. They revealed birth and death certificates are provided free of cost, given registration was done within 21 days of the event. Moreover, delayed registration is allowed on submission of the affidavit, late fine and block and district level officers' approval. We found most of the staff perceived timely and complete birth and death registration is crucial for generating accurate vital statistics for policymaking. However, half of the total CRS staff interviewed did not read the CRS manual.

"I enter birth and death data into the system. A few times I feel difficult to assign correct codes in the birth and death registration forms due to unavailability of latest CRS manual in the office." (a CRS staff, Patna**)**

More than half of the total staff interviewed (10 out of 15) mentioned that targets were provided for birth and death registration by the office of Registrar General of India~~state headquarter~~. The target for registration coverage is higher for births (>90%) than death registration (80%). These targets are the estimated number of birth and death to be registered by the CRS staff in a particular month. CRS manual shows the mathematical formula for calculating such a target.

They also expressed that most of the medical attendant was not skilled in recording the cause of death.

"I received a monthly target of birth and death registration by the state headquarter. I don't know the mathematical formula for calculating the target. Also, I don't believe in such a target as institutional birth data are transferred directly to the Nagar Nigam and then district headquarter and not shared with me. Many times, the target is unreliable and difficult to achieve it". (a CRS staff, block office, Vaishali).

2. Administrative and technical challenges

Despite the government's announcement of the CRS portal for birth and death registration at a real-time basis in all the registration units, we found 1 out of 4 block offices of Patna, and 3 out of 4 block offices of Vaishali were not equipped with modern technologies such as computers, printers, photocopier and specific CRS software. Irregular electric supply and no separate department for CRS were observed.~~ Most of the staff interviewed was associated with other departments~~. ~~However, they~~ We also found many staff of other department performed the duties of birth and death registration besides their departmental work.

"I am often given duty as an invigilator during the state's secondary board examination and other examination for screening of the candidates for public sector's jobs. I am also directed to assist officers in the conduct of municipalities or the state-level election." (a block-level officer, Patna)

This study found an a~~a~~nganwadi s~~s~~evika performs multiple tasks such as providing pre-natal and post-natal care, assistance during birth delivery, and birth and death registration. They also teach children enrolled at their centre and serve mid-day meals to them. The pressure of performing multiple tasks limited their role as a sub-registrar and affected the quality of registration.

Formatted: Font: Not Bold, Not Italic

"Block officers assign multiple tasks to me. I am required to run an anganwadi centre where I teach and serve mid-day meals to the children . I also register birth or death occurs in this area." (an a~~a~~nganwadi s~~s~~evika, Vaishali)

Furthermore, there was no regular training provided to the CRS staff. We found most of the CRS staff did not receive training in last one year. ~~. A few medical staff associated with CRS receive training on the recording of the cause of death and online registration.~~ Anganwadi s~~s~~evika was not invited in the block level meeting since last year. Besides, they were not provided with a birth and death registration forms and honorarium on time. In addition, there was also no adequate fund available for the periodic training and workshops for the staff.

"Block-level officers don't call me in the review meeting. I think monthly, or quarterly review meeting is required for solving problems encountered during birth and death registration." (an a~~a~~nganwadi s~~s~~evika, Patna)

"I have not received any training in the last two years. I attended a training program on organising workshops on awareness on birth and death registration benefits two years back. I need training on how to operate CRS portal on a computer system." (a block-level officer, Patna)

There was no regular supervision at the block level or community level by the district or state level officers. Additionally, there was no strict implementation of the RBD Act, and the officers did not take any steps for improving the registration level in recent times, particularly death registration. We found health institution does delayed registration; however, it is not allowed as per official guidelines. We found poorer functioning of the registration system in the block offices of Vaishali as compared to the block offices of Patna.

3. Staff's suggestions to improve birth and death registration level

Most staff (10 out of 15) interviewed revealed that regular awareness programs on birth and death registration benefits could increase registration levels. Posters and banners exhibition during most celebrated festivals like Durgapuja, Chhat Puja, Ramnavmi procession, Harihar Kshetra Mela (Sonepur cattle fair which attracts visitors from all over Asia) was widely suggested by the officials and staff. A few staff (5 out of 15) ~~-~~told mobile registration in hard-to-reach areas, or the villages far from registration centres could improve birth and death registration levels.

Further, medical personnel associated with CRS revealed that the birth and death reporting had increased after CRS' association with health facilities. ~~Civil authorities should expand birth and death registration at PHCs in rural areas.~~ Besides, the system manager, an assistant director, and

block-level officers revealed that adequate awareness campaign on importance of death registration is needed to ~~mandatory burial or cremation permits might~~ increase death reporting. In addition, they suggested that adequate funds should be provided for the smooth functioning of the system.

We also interviewed two CRS staff over telephone after third wave of Covid (January 2022) in India. They revealed that they did not achieve the birth and death registration level target due to shortage of staff. Majority of staff were appointed in C~~c~~ovid management duty. Therefore, the system failed to record birth and death on time during the pandemic.

Discussion

The present study investigated CRS's performance barriers in a poor state of India where birth and death registration is low. In 2012, there had been lower recording of birth and death. Again, there was slowdown in registration due to revamping of system in 2016 from paper-based registration to online system (CRS portal) for registration of vital events. Moreover, in order to accelerate the vital statistics quality and coverage and to create the awareness among the public, a descriptive analysis was published in the quarterly report of Directorate of Economics & Statistics namely "Bihar Sankhyaki Darpan". The message on benefits of birth and death registration were also telecasted from Doordarshan Patna to attract the attention of rural and urban people towards registration [33].

The inadequate knowledge and attitude of community members on birth and death registration procedures and benefits are barriers to registration levels. Previous studies documented unawareness among people is a significant challenge in birth and death registration [2,23,31]. In our study area, knowledge on registration was not a barrier, yet we found a discouraging attitude, particularly to death registration, contributed to incomplete CRS in the study area. Perception of little benefits of death reporting led to lower death registration in Bihar. The inadequate knowledge and perceptions of low benefits of ~~related to~~ birth and death registration limited the coverage of CR~~V~~S in lower and middle-income countries [30,34~~3~~].

Formatted: Font: (Default) Times New Roman, 12 pt

Formatted: Font: (Default) Times New Roman, 12 pt

Formatted: Font: (Default) Times New Roman, 12 pt

Formatted: Font: (Default) Times New Roman, 12 pt

Formatted: Font: (Default) Times New Roman, 12 pt

Most people reported their child's birth to civil authorities because there are financial and social benefits associated with birth certificates. The government of Bihar encourages birth registration and providesprovides financial assistance to female children after their birth registration to a female child after her birth registration [354]. Moreover, birth certificate is required for child’s school enrolment, vaccination and his social security [4,5]. Unlike reporting a child's birth, reasons for lower reporting for a person's death are not widely known. Consistent with previous studies, our study also documented a lower registration of infant and female deaths [24,25,27]. According to our participants, a lower death registration among females and children is due to a lack of legal necessity and no immediate financial or property-related benefits. We also observed that the presence of social stigma for premature death is negatively impacting death registration coverage. A lower reporting of infant deaths resulted from traditional beliefs such as an infant death as the sin of parentsparent’s previous birth. For burial permission, reporting -registration of death to civil authority was not -requiredmandatory. Therefore, there is a lower priority for death registration among the participants. Underreporting of infant deaths that happen outside of facilities may lead to underestimating infant mortality.

In addition, differential reporting by sex will lead to insufficient quality data on sex differences in mortality rates [36].. Higher death registration of men could be associated with their engagement with the formal employment sector. Generally, family members are required to submit a death certificate to an employer to obtain social safety benefits after the death of a working member of the household. However, in our study areas, most of the respondents were engaged in the informal sector such as farming, construction of houses, a small business etc. that seldom provide social benefits to their families. Financial incentives for performing funeral activities and minimal financial assistance to deceased families may improve death reporting [375].

Even birth and death that occurred in health facilities was not recorded due to lack of dedicated staff and staff’s negligence. shortage of registration forms. Public facilities record all birth and death but don't update on the CRS portal until birth and death certificates are requested by family members. A study documented a similar argument for lower reporting of institutional birth -and death in Bihar [38]. No sNo strict implantation of the law is the primary reason for lower coverage of birth and death in Bihar. The previous study also revealed strict implementation and

periodic audit of death registration is required [9,31,~~36~~38,39]. ~~In many countries, birth and death registration is mandatory, and a fine is strictly imposed for not reporting the event within the prescribed time [2,37].~~

Formatted: Not Highlight

This study highlighted poor services at the CRS offices such as ~~which includes a complex registration form~~a~~,~~ longer waiting time at the registration centres and not providing the certificates on time. In addition, the demand of bribes by CRS staff is discouraging for the participants not to report birth or death. Indirect opportunity costs such as travelling costs to the registration centre, loss of wage for a day were barriers to birth and death registration. Mobile registration in far-flung areas, reducing documentation work of applicants and assisting people in generating identity cards from concerned agencies would increase birth and death registration coverage. Moreover, awareness on specific cases like the adoption of a child and the death registration of a missing person should also be provided. a~~A~~nganwadi sevika~~sha workers~~, local leaders and district officials could be engaged in awareness programs with a minimal level of training to decrease negative impact of the customs or traditional beliefs on birth and death registration~~with a minimal level of training~~.

A knowledgeable and well-trained staff is essential for effective CRS [7]. This study showed staff had a good understanding of birth and death registration law and registration procedures. However, the lack of adequate training on the use of the CRS portal was a barrier to recording vital events. Department lack dedicated staff deputed solely for CRS work. Multiple tasks overburdened CRS officials led to compromise with the quality of vital statistics. The previous study also documented lack of dedicated staff, lack of training on the use of computer systems for recording birth and death was barriers to CRS functioning [9, 31]. Refresher training is also essential for updating and solving the fields' problems [7,38]. ~~[7].~~

Commented [D2]: Numbering is missing

Regular monitoring of ~~Establishing a community-based birth and death registration unit, i.e.,~~ registration unit at primary and community healthcare, may ensure accessibility and improve birth registration level. Previous studies documented urbanisation and access to healthcare facilities are associated with higher birth registration [9,27,31,40~~38~~]. An improvement of health facilities in Bihar is needed to improve coverage of birth and death registration. According to

National Family Health Survey-2019-20, Bihar has one of the lowest maternal and child health care service utilisation within the country [41~~39~~].

Telephonic interview with CRS staff reveal that during the Covid, the system failed to achieve the target of birth and death registration as there is shortage of CRS staff. Besides, staff were appointed on Covid management duty such as awareness program, monitoring and inspection of free supply of food, medicines and other healthcare items.

This study suggests some crucial points to improve birth and death registration levels. First~~ly~~, recurring awareness programs on birth and death registration benefits for CRS staff and community members particularly, rural residents. Customs or traditional beliefs led to lower reporting of vital event, should be changed with adequate awareness campaign. ~~Second, linkages of health facilities to CRS should be expanded to all PHC in rural areas. Third, mandatory permit for burial or cremation and minimal financial support for funeral rites.~~ Second~~Fourth~~, there is a need to improve accessibility to registration centres and the use of ICT to speed up the CRS work. Third, ~~Lastly,~~ adequate funds, dedicated staff and regular monitoring and auditing of the vital statistics may improve the registration level. Fourth, standardized operating protocol should be developed for uniform processes in the system because many registration offices are not aware of CRS manual and how to use CRS portal effectively. Fifth, state-wise registration target are set by Registrar General of India, however it is based on crude birth and death rate. We recommend state, district and sub-district level target needs to be worked out using some robust scientific methodology. The aspect of migration, live birth reported by health management information system should be taken into consideration while estimating registration target.~~CRS must be seen as a priority by political leaders and stakeholders.~~

Despite a comprehensive analysis, this study has some limitations. This study covers only two districts of Bihar; therefore, generalisation could be made for the states or districts with a similar development level with care. We did not show comparison study with CRS operations in other states with similar level of development due to limited literature. We did not collected data by ~~caste~~socio-economic background (such as education, wealth, religion or castes) of study participants; therefore, ~~caste wise~~such analysis cannot be done in this study. ~~Also~~In addition, this study is mainly focused on barriers at the operational level and not assessed the quality of

vital statistics. Moreover, we did not check the birth and death certificate owned by respondents, so there could be under-reporting or over-reporting of birth and death registration due to recall bias. P~~Besides, p~~ossession of a birth certificate is considered socially desirable, so there might be bias reporting of birth registration.

Conclusions

Delayed or absent birth records could limit the government's ability to understand the population's needs and are associated with long-term health care use and outcomes. Lack of death registration and evidence of disparities in registration by sex could lead decision-makers ~~not to~~ target services inappropriately and lead demographers to make inaccurate estimations of trends. While support for those collecting this information is crucial, addressing socio-cultural beliefs and incentives for the death registration of certain people (infants, women) is also critical. Adequate awareness campaign is required to increase birth and death registration level in Bihar.~~The provision of mandatory registration of death before burial or cremation of deceased persons could improve the death registration level.~~

Acknowledgements

The authors would like to acknowledge study participants who co-operated and provided their valuable time for the in-depth interview and FGD. Besides, we are thankful to all the Ambedkar Library staff, Jawaharlal Nehru University, New Delhi, for providing access to Journals

Authors Contributions

Conceptualisation: Nandita Saikia (NS)

Data curation : Krishna Kumar (KK)

Formal analysis: Krishna Kumar

Methodology: Nandita Saikia & Krishna Kumar

Supervision: Nandita Saikia

Writing- Original draft: Krishna Kumar

Writing- review & editing: Nandita Saikia & Nadia Diamond-Smith

References

1. UNICEF. United Nations Children's Fund. Every Child's Birth Right: Inequities and trends in birth registration. UNICEF, New York. 2013. https://www.un.org/ruleoflaw/files/Embargoed_11_Dec_Birth_Registration_report_low_res.pdf Accessed on 2nd Oct 2021.
2. Office of Registrar General of India (ORGI). Vital Statistics of India based on the Civil Registration System 2019. New Delhi. 2021. https://censusindia.gov.in/2011-Common/CRS_2019/CRS2019_report.pdf Accessed on 25th Sep 2021.
3. UNICEF. Birth registration for every child by 2030: are we on track? UNICEF. 2019. https://reliefweb.int/sites/reliefweb.int/files/resources/Birth-registration-for-every-child-by-2030-brochure.pdf Accessed on 25th Nov 2020.
4. AbouZahr C, Cleland J, Coullare F, Macfarlane SB, Notzon FC, Setel P, Monitoring of Vital Events (MoVE) writing groups. The way forward. The Lancet. 2007; 370(9601): 1791-99. https://doi.org/10.1016/S0140-6736(07)61310-5
5. Duryea S, Olgiati A, Stone L. The under-registration of births in Latin America. 2006. IDB Working Paper No. 458. https://dx.doi.org/10.2139/ssrn.1820031
6. Setel PW, Macfarlane SB, Szreter S, Mikkelsen L, Jha P, Stout S, Monitoring of Vital Events (MoVE) writing groups. A scandal of invisibility: making everyone count by counting everyone. The Lancet. 2007; 370(9598):1569-77. https://doi.org/10.1016/S0140-6736(07)61307-5
7. United Nations. Principles and recommendations for a vital statistics system. United Nations. 2014. https://unstats.un.org/unsd/demographic/standmeth/principles/m19rev3en.pdf Accessed on 25 Sep 2021
8. WHO. Civil registration: why counting births and deaths is important. WHO. 2014 https://www.who.int/news-room/fact-sheets/detail/civil-registration-why-counting-births-and-deaths-is-important Accessed on 1st Mar 2021.

9. Oung MT, Richter K, Prasartkul P, Aung Y, Soe KT, Tin TC, Tangcharoensathien V. Reliable mortality statistics in Myanmar: a qualitative assessment of challenges in two townships. BMC Public Health. 2019;19(1):1-10. https://doi.org/10.1186/s12889-019-6671-y
10. Nojilana B, Groenewald P, Bradshaw D, Reagon G. Quality of cause of death certification at an academic hospital in Cape Town, South Africa. South African Medical Journal. 2009; 99(9). https://hdl.handle.net/10520/EJC69546
11. Rampatige R, Gamage S, Peiris S. Assessing the reliability of causes of death reported by the Vital Registration System in Sri Lanka: medical records review in Colombo. HIMJ. 2013; 42(3):20–28. https://doi.org/10.1177%2F183335831304200302
12. Australia Indonesia Partnership for Justice (AIPJ). Centre on Child Protection. Indonesia's missing millions. AIPJ baseline study on legal Identiy. 2014. http://www.cpcnetwork.org/wp-content/uploads/2015/02/AIPJ-PUSKAPA-BASELINE-STUDY-ON-LEGAL-IDENTITY-Indonesia-2013.pdf Accessed on 26 Mar 2021
13. UNICEF. The "Rights" Start to Life: A Statistical Analysis of Birth Registration. UNICEF. 2005. https://www.unicef.org/spanish/protection/rights_to_life.pdf
14. Zeng X, Adair T, Wang L, Yin P, Qi J, Liu Y, Liu J, Lopez AD, Zhou M. Measuring the completeness of death registration in 2844 Chinese counties in 2018. BMC medicine. 2020; 18(1):1-1. https://doi.org/10.1186/s12916-020-01632-8
15. Saikia N, Kulkarni PM. Data for Research into Health Inequality in India. The Economic and Political Weekly. 2016; 51(26):111-6.
16. Saikia N, Kulkarni PM. An assessment of India's readiness for tracking SDG Targets on health and nutrition. ORF Occasional Paper. 2018;108. https://www.orfonline.org/research/an-assessment-of-indias-readiness-for-tracking-sdg-targets-on-health-and-nutrition/
17. United Nations. Sustainable Development Goals: Goal 3. United Nations. https://www.un.org/sustainabledevelopment/health/ (accessed on 2 October 2021.)
18. International Institute for Population Sciences (IIPS) and ICF. National Family Health Survey (NFHS-4), 2015-16: India. Mumbai: IIPS. 2017. http://rchiips.org/NFHS/NFHS-4Report.shtml
19. Office of Registrar General of India (ORGI). Vital Statistics of India based on the Civil Registration System 2014. New Delhi. 2016. https://www.censusindia.gov.in/2011-Documents/CRS_Report/crs2014_final.pdf Accessed on 21 Sep 2020.

20. Office of Registrar General of India (ORGI). Vital Statistics of India based on the Civil Registration System 2017. New Delhi. 2019. http://crsorgi.gov.in/web/uploads/download/CRS_report_2017_20192020.pdf Accessed on 29 Sep 2020.
21. Office of Registrar General of India (ORGI). Vital Statistics of India based on the Civil Registration System 2018. New Delhi. 2020. https://censusindia.gov.in/2011-Common/CRS_2018/crs2018_20072020.pdf Accessed on 2 Ooctober 2021.
22. Hill K, Lopez AD, Shibuya K. Interim measures for meeting needs for health sector data: births, deaths, and causes of death. The Lancet. 2007;370(9600):1726-35. https://doi.org/10.1016/S0140-6736(07)61309-9
23. Li S, Zhang Y, Feldman MW. Birth registration in China: practices, problems and policies. Population Research and Policy Review. 2010;29(3):297-317. https://doi.org/10.1007/s11113-009-9141-x
24. Gupta M, Rao C, Lakshmi PV, Prinja S, Kumar R. Estimating mortality using data from civil registration: a cross-sectional study in India. Bulletin of the World Health Organization. 2016;94(1):10. https://dx.doi.org/10.2471%2FBLT.15.153585
25. Kumar, G. A., Dandona, L., & Dandona, R. (2019). Completeness of death registration in the Civil Registration System, India (2005 to 2015). *The Indian journal of medical research*, *149*(6),740-47. https://dx.doi.org/10.4103%2Fijmr.IJMR_1620_17
26. Rao C, Gupta M. The civil registration system is a potentially viable data source for reliable subnational mortality measurement in India. BMJ Global Health 2020;5:e002586. http://dx.doi.org/10.1136/bmjgh-2020-002586
27. James K S, Kavita N, George A. Preliminary assessment of the quality of civil registration system in Kerala, Odisha and Rajasthan. UNFPA. 2013. http://india.unfpa.org/sites/default/files/pub-pdf/ReportonCRS-Final.pdf
28. Rao N, Kaul V. India's integrated child development services scheme: challenges for scaling up. Child Care Health Dev. 2018; 44(1):31–40. https://doi.org/10.1111/cch.12531 PMID: 29235171
29. Gopalakrishnan L, Buback L, Fernald L, Walker D, Diamond-Smith N, in addition to The CAS Evaluation Consortium. Using mHealth to improve health care delivery in India: A qualitative examination of the perspectives of community health workers and beneficiaries. PLoS ONE. 2020; 15(1): e0227451. https://doi.org/10.1371/journal.pone.0227451

30. Haider MM, Alam N, Ibn Bashar M, Helleringer S. Adult death registration in Matlab, rural Bangladesh: completeness, correlates, and obstacles. Genus. 2021; 77 https://doi.org/10.1186/s41118-021-00125-7

31. Rane TM, Mahanta TG, Islam S, Gogoi PP, Gogoi B. Civil registration system (CRS) for birth and death registration in Assam–a rapid assessment. Clinical Epidemiology and Global Health. 2020; 8(1):117-22. https://doi.org/10.1016/j.cegh.2019.05.006

32. Braun V, Clarke V. Using thematic analysis in psychology. Qualitative research in psychology. 2006;3(2):77-101.

33. Office of Registrar General of India (ORGI). Vital Statistics of India based on the Civil Registration System 2012. New Delhi. 2015. https://censusindia.gov.in/2011-Documents/CRS_Report/CRS_Report2012.pdf

~~33.~~34. Fisker A, Rodrigues A, Helleringer S. Diferences in barriers to birth and death registration in Guinea-Bissau: Implications for monitoring national and global health objectives. Tropical Medicine and International Health. 2019;24(2),166–174. https://doi.org/10.1111/tmi.13177

35. Women Development Corporation. Mukhyamantri Kanya Suraksha Yojna. Bihar. https://wdc.bih.nic.in/MKSYDetails.aspx Accessed on 2 October 2021.

~~34.~~36. Adair T, Gamage USH, Mikkelsen L, et al. Are there sex differences in completeness of death registration and quality of cause of death statistics? Results from a global analysis. BMJ Global Health 2021;6:e006660. http://dx.doi.org/10.1136/bmjgh-2021-006660

~~35.~~37. Garenne M, Collinson MA, Kabudula CW, Gómez-Olivé, FX, Kahn K, Tollman S. Completeness of birth and death registration in a rural area of South Africa: The Agincourt health and demographic surveillance, 1992–2014. Global Health Action. 2016; 9(1), 32795. https://doi.org/10.3402/gha.v9.32795.

38. Kumar D, Pandey BK, Kumar Ajit, Basu JK. Civil Registration in Bihar: Strengths and Challenges. Demography India. 2021.

~~36.~~39. Patel AB, Rathod H, Rana H, Patel V. Assessment of Medical certificate of medical certificate of cause of death at a new teaching hospital in Vadodara. Natl J Commun Med. 2011;2(3):349–353.

~~37. Health System Research Institute (HSRI). Review of National Civil Registration and Vital Statistics Systems: A case study in Thailand. Bangkok: Thai Health Information Standard Development Centre (THIS). 2015.~~

Formatted: Font: Font color: Text 1

Formatted: Indent: Left: 0.5", No bullets or

Formatted: Font color: Text 1

Formatted: Adjust space between Latin and Asian text, Adjust space between Asian text and numbers

Formatted: Font color: Text 1, Pattern: Clear (White)

~~38.~~40. Fall A, Masquelier B, Niang K., Ndiaye S, Ndonky A. Motivations and barriers to death registration in Dakar, Senegal. *Genus*.2021;*77*(1), 1-20. https://doi.org/10.1186/s41118-021-00133-7

41. Ministry of Health and Family Welfare & International Institute for Population Sciences (IIPS). Bihar Fact Sheet. National Family Health Survey (NFHS-5) 2019-20. India. Mumbai: IIPS. 2021. http://rchiips.org/nfhs/NFHS-5_FCTS/FactSheet_BR.pdf

~~39.~~

Formatted: Font: Bold, Font color: Text 1

Formatted: Normal, No bullets or numbering

Supporting information

S1 Text. Guide for KII and FGD.

~~**S2 Text 2.** Names of blocks of Patna and Vaishali.~~

~~**S3 Text 3.** COREQ checklist.~~

S1 Table. Rate (%) of health and education indicators in the Bihar and India, 2015-2016.

S2 Table. Names of blocks of Patna and Vaishali.

S3 Table. Estimates of birth and death registration level by districts of Bihar

S3 Text 3. COREQ checklist.

Formatted: Font: Not Bold

Table 1. Demographic profile of the study participants

	Mean age (in years) & (range)	Study Area				Number of respondents	Method of data collection
		Patna		Vaishali			
		Male	Female	Male	Female		
**Assistant Director**	50	1	-	-	-	1	KII
**System Manager**	35	1	-	-	-	1	KII
**BSO**	41 (40-42)	1	-	1	-	2	KII
**Anganwadi Sevika**	44.3 (40-50)	-	2	-	2	4	KII
**Medical officer**	52.2 (45-62)	-	2	1	-	3	KII
**Ward Parsad**	29.5 (24-35)	1	-	1	-	2	KII

**Nagar Nigam**	36 (30-42)	1	-	1	-	2	KII
**Community members**	35.6 (17-62)	30	13	25	22	90	FGD

S1 Table. Rate (%) of health and education indicators in the Bihar and India, 2015-2016.

**S. No.**	**Indicators**	**Bihar**	**India**
**1**	**Total population (2016) (million)**	113[a]	1324
**2**	**SCs Population (%)**	15.91	16.63
**3**	**STs Population (%)**	1.28	8.60
**4**	**Proportion Hindus (%)**	82.69	79.80
**5**	**Proportion Muslims (%)**	16.87	14.23
**6**	**Total Fertility Rate**	3.14	2.18
**7**	**Crude Birth Rate**	27.00	19.00
**8**	**Under-five mortality rate (per 1000 live births)**	58.00	50.00
**9**	**IMR (per 1000 live births)**	48.00	41.00
**10**	**Twelve or higher years of schooling among household populations age six and above (%)**	6.80	13.70
**11**	**Children who received immunisation from Anganwadi sevika (%)**	41.10	39.80
**12**	**Children who received health check-ups from Anganwadi sevika (%)**	32.80	31.20
**13**	**Institutional birth (%)**	64.00	79.00
**14**	**Birth registration level (%)**	60.90	79.90
**15**	**Net State Domestic Product Per Capita (2018-19) (US$)**	1964.00	640.00

Data Source: **For Health indicators**: NFHS, 2015-16**; For SCs, STs, Religion**: Census 2011**; For Net State Domestic Product Per Capita**: MOSPI**; For Population**: United Nations.[a]

Estimated population of Bihar (2016) = Estimated population of India (UN, 2016) * proportion of the population of Bihar (8.57%) to India (census 2011).

Reply to respected reviewers.

Reviewer #1: This is a good piece of scientific work addressing a very important yet neglected issue. Good efforts have been put together to develop this manuscript though it needs some major revisions.
Major points:

1. Line Nos 121-129: Please make the study rationale more clear

Reply- We have revised the manuscript in light of your suggestion. Please see line no. 121-130.

2. Figure 1:
a. How are the deaths at 'home' in 'urban' areas registered? Missing in the diagram.

Reply- We have revised the diagram in light of your suggestion. Please see page no.5, fig. 1

b. How are the deaths in 'hospitals' in rural areas registered? missing in the diagram.

Reply- We have revised the diagram in light of your suggestions. Deaths in Hospitals are registered by the head of the institution (Chief Medical Officer). Please see page no.5, fig 1.

c. Is ASHA/ANM playing any role in death registration as being currently used in Punjab and Haryana? if yes, then mention, please. Who are the designated key informants to whom family members can report FIR of an event in both urban and rural areas?

Reply- In Bihar, ASHA/ANM does not play any role in birth and death registration.

We have revised the manuscript now. Please see page no. 5, fig 1

d. At the district and state level, which department is responsible for births and deaths registration? Is it the Health department like in Haryana and Punjab? These points are not coming out explicitly from the diagram itself!!

Reply- Registrar at Nagar Nigam, District registrar office, and Chief medical officer of district-level hospitals are mainly responsible for birth and death registration. At the state level, Chief registrar, Joint Chief Registrar, and Dy. Chief Registrar (Vital Statistics) is responsible for birth and death registration. We have revised fig 3. (Page no. 7)

3. Please mention three things in each box of Figure 3: Designation of the person under CRS, name of the department the person holds, and his/her designation in that department. Example: Like you have mentioned Chief Registrar is the Director of Statistics and Evaluation but of which department???? Please elaborate

Reply- Chief Registrar is the Director of the Directorate of Statistics and Evaluation belonging to the Planning and Development Department in Bihar. We have revised fig 3 in light of your suggestion. Please see fig3, Page no. 7

4. Did you consider the levels of birth and death registration of district and block as selection criteria for sampling units because that might give a better picture of reasons.

Reply- We observed district-level death registration level as selection criteria in addition to urbanization rate. We purposively selected a district with higher death registration and a district with lower death registration. Furthermore, four blocks are chosen randomly from each district. We have revised the write-up now. Please see line no. 153-159.

5. The methodology is indicating that the study was conducted among the rural aspect of CRS as none of the study participants represented the urban population or municipal corporation. This has undermined the study findings to rural areas only. I feel you need to explain this as a study limitation and provide a valid reason for not including the urban representation.

Reply- We also included considerable study participants from urban areas. We have presented the methodology more clearly now. Please see line no. 170-177.

6. In the methodology section, it's important to mention the levels of birth and death registrations of the two districts you have selected. and compare it with other districts.

Reply- We have included the death registration level of two selected districts (Patna and Vaishali) in methodology (lines no. 157 & 159). We have provided a supplementary table showing birth and death registration levels by the districts of Bihar. Please see supplementary file S3 Table.

Minor revisions:

1. Please use one terminology throughout either CRS or CRVS in the introduction section

Reply- We have revised the manuscript in light of your valuable suggestions. We are using CRS throughout the manuscript.

2. Expand ORGI in the caption of Figure 1

Reply- We have revised the manuscript. We have written a different title now. ” ***Fig-1 Flow chart of birth and death registration in Bihar” (Please see page no. 5)***

3. Please correct the spelling of 'Anganwadi sevika' throughout the manuscript

Reply- We have corrected the spelling of Anganwadi sevika in the revised manuscript.

4. Mention in the data collection part of the methodology section about the total number of FGDs done

Reply- We have included the total number of FGD done in the revised manuscript. Please see line no.- 183

5. Mention in the data collection part of the methodology section about the total number of KII done

Reply- We have included the total number of KII done in the revised manuscript. Please see line no. 183

6. Line nos. 290-93 not clear

Reply- We have revised the manuscript now. Please see line no. 248-253

7. Please provide supporting data for statement no. 528-529

Reply- We have included the supporting data now. Please see line no. 505

8. Please provide supporting data for statement no. 338-39

Reply- We have found lower reporting for death, particularly, infant and female death, in Bihar. We provided the transcript along with this manuscript. A study also showed death registration coverage is poor in Bihar (Reference no. 38 in the manuscript)

9. Please add references for lines 182-186

Reply- We have included a supplementary table (S3 table) in support of lines 182-186.

10. In line no. 556-57, you have mentioned that birth and death registration is not being done at sub-center or village level? Is it not community-based as of now? Review the lines

Reply- In rural areas, birth and death occur at home are registered by Anganwadi sevika within 21 days of the event. In urban areas, birth and death occur at home is registered by registrar at municipal corporation and block offices. Institutional birth and death is registered by designated health staff at health centers within 21 days of events. Please see fig 1, page no. 5. We have revised the manuscript now.

11. You have mentioned in line 567 that all PHCs are not linked to CRS. Is this statement true? Please review.

Reply- We have found many PHC are not linked to CRS due to lack of adequate logistics and lack of dedicated staff or negligence by the medical officer. A recent paper also showed registration are missed in PHC due to negligence (Kumar et al., 2021)

Reviewer #2: Review: Performance barriers of Civil Registration System in Bihar: An exploratory study

This is an interesting exploratory study, relying on interviews with government officials and rural residents, which documents the functioning of the civil registration system in Bihar before the pandemic. Given the importance of understanding and improving the civil registration system, such studies are urgently needed. I felt that the study should have been more in-depth. Interviews with CRS officials and with community members could have been more detailed, and the description of the overall state of the CRS in Bihar, despite the authors' attempts, is still quite incomplete. However, given the paucity of scholarship on the civil registration system in India, specially of a qualitative nature, this paper is welcome. I have some minor suggestions, which I hope would be helpful for the authors in revising their work.

1. The authors motivate their paper saying that it is important to study the civil registration system in Bihar because of its poor development indicators in general, and the fact that its CRS is estimated to record the lowest proportion of deaths and births. However, although the authors provide some hints at why Bihar's CRS performs so poorly, we do not get an explicit discussion of why precisely Bihar lacks even other northern Indian states, such as Madhya Pradesh or Rajasthan, which have higher registration coverage than Bihar. In this, the state of Bihar and Uttar Pradesh are way behind all the other states in India, and it is not clear why this is the case. To truly understand this, a comparative design is needed. Perhaps the case is that the lack of linkages between health systems and the CRS which the authors document, or the lack of computerization in Bihar explains why it lags behind other Indian states (even similarly poor ones). My suggestion to the authors is to consider this line of thinking carefully. They can see what the extant literature says about other poor Indian states (they may find looking at the annual CRS reports at the state level and the national level useful here) about CRS being so poor in Bihar. They should also note some lines of thinking for future researchers in the discussion section, as well as perhaps a limitation that they have not adequately addressed these questions.

Reply- There are limited studies on CRS at state levels, particularly in Bihar. We could not locate any study examining why CRS is poor in Bihar. Although we welcome the reviewer's comment on a comparative picture of Bihar/UP with MP or Rajasthan, it is beyond the scope of the current study. Therefore, a comparative study cannot be done for the present study as it collected information of Bihar only. We may explore this research gap in future studies. We have included these lines in the limitation section of this manuscript. Please see line no. 560-561.

2. Related to this point, we also don't get a sense of the changes in the Civil Registration System before the pandemic in Bihar. Existing patterns, both from the data that has been accessed by journalists during the pandemic period (for instance

see https://www.indiacovidmapping.org/reports/mortality/BiharFactsheet.pdf), as well as annual estimates before the pandemic suggest improvements in the Civil Registration System in Bihar. How did these improvements came about? If the authors have information on this, it would be extremely valuable. Similarly, what happened to Bihar's civil registration system during the pandemic? Answering this likely requires fresh data collection. If that is not feasible, then that should be noted in the discussion. If it is possible to call CRS staff on phones and ask some of these questions, then that would be quite valuable. If this is not possible, then perhaps the authors can consider this as a future research endeavor.

Reply- We have included information regarding development of CRS in Bihar before pandemic. Also, we added what happen to CRS in Bihar during the pandemic in the revised manuscript. Please see line no. 452-455.& 459-466.

3. At a large number of places, I felt that the authors have policy recommendations that were not supported by the information they present. In some cases, their recommendations can actually be harmful. For instance, the authors recommend "mandatory burial or cremation permits." Instead of improving registration, I can see how this kind of procedure would cause even more problems in a place like Bihar. For instance, people will have to wait to get a death registered and could then only approach burial or cremation grounds? Instead of this, what would work well is a "notification" and "proof" system: burial grounds can notify CRS authorities about a death, and the family could have a slip which would help as a proof of death. These linkages exist in other states, especially with healthcare facilities. Public hospitals can in fact issue death certificates in many states, and private hospitals issue a hospital death certificate (or birth certificate) which can be taken to a registrar to get a death registered.

I was also uncomfortable with the recommendation of fines (lines 533-534). First, the authors already say that there is a fine of Rs. 10 to register a death after 21 days. Worse, the procedure described here to register a death after 21 days looks like a nightmare, with visits to far off offices. More fines when clearly it is the government system that is lacking is a bit counterproductive. The authors should remove this recommendation. In any case, this does not follow from the data presented by the authors.

Reply- We have revised the manuscript in light of your valuable suggestions. We have deleted mandatory registration burial or cremation and fine for late registration.

4. I found the sentence "there was no strict implementation of the birth and death registration law" vague. Which aspects of the law were not strictly implemented, and how was this ascertained?

Reply- We found health institution does delayed registration; however, it is not allowed as per official guidelines. These event should be registered by Block Development Officer or District Statistical Officer in such cases. A similar problems were also shown by a previous literature (Kumar et al; 2021).We have included this sentence in revised manuscript. Please see line no. 433-434.

5. I was not convinced by the literature cited that "lack of birth registration is associated with school dropout …" and so on. It is quite likely that this is just selection, for instance poor children are more likely to drop out and poor children are less likely to have had their births registered. I would suggest dropping this citation.

Reply-We tried to show the association of unregistered birth and school dropout. We don't intend to show absence of birth certificate is a causal factor of the school drop-out. However, we have reframed the sentence now. Please see line no. 91-92

6. The authors cite the CRS report to report that 7 percent of births and 8 percent of deaths were not registered in 2019. Although this is an official estimate, it is likely an over-estimate. There are multiple reasons for this, among them the fact that the 2019 CRS deaths in this report are compared to 2018 SRS. Other reasons include the fact that the SRS may under-estimate the crude death rate. This line of thinking is explored in detail here (appendix 1: https://www.medrxiv.org/content/10.1101/2021.09.30.21264376v1). I leave it to the authors to decide how best to convey the uncertainty in the true rate of registration completion. Perhaps the authors can say "according to official estimates …".

Reply- We have written "According to official statistics", line no. 95-96.

7. In figure 1, which provides the organization of the CRS, I was surprised that the Panchayat was not mentioned anywhere in the hierarchy. In figure 3, the panchayat sevak is mentioned as a registrar. In some other northern states, such as Madhya Pradesh, the bulk of rural death and birth registration happens at the Panchayat level. Is it the case that this does not happen in Bihar? In Madhya Pradesh, I have seen functioning computers in Panchayat offices which can register deaths. In which case, this may be the reason why death registration is so poor in Bihar – the lack of panchayat registration. The authors could perhaps explore this more, and correct figure 1 if necessary. If the panchayat is not a functional place to register deaths, then this is actually an important policy suggestion, to improve registration and computerization at the Panchayat level.

Reply- Panchayat secretary office registers birth and death after 21 days of the occurrence of event in rural areas in Bihar. I have revised the fig 1. Please see page no. 5

8. Figure 1 claims that an annual report at the "state level is published by the chief registrar" and also that "Annual report at India and state level is published by ORGI". This is confusing, and likely the latter sentence needs modification.

Reply- Annual report at India level is published by ORGI, New Delhi. In Bihar, state level report is published by Directorate of Economic and Statistics, Department of planning and development. We have changed the fig 1 now. (Please see page no .5)

9. I thought that the bit about people not reporting infant deaths because it is considered a sin of parents in previous birth was quite revealing. The state government should consider potentially challenging this belief, and this also is a policy recommendation that flows from the findings from the paper. In addition, given the levels of awareness that the authors document, perhaps more information campaigns are also worth recommending.

Reply- We have revised the manuscript in light of suggestions. Please see line no. 544-45

10. Similarly, I was surprised by the sentence that "Public facilities record all birth and death but don't update on the CTS portal until birth and death certificates are requested by family members." The authors do not present evidence for it. This is quite important, and also

deserves to have highlighting from the perspective of policy recommendation that the paper generates.

Reply- We observed health staff don't update registered data from registers to the CRS portal until birth and death certificates are requested by family members. A study documented a similar problem (Reference no. 38). We recommended for proper monitoring of all registration units. Please see line no. 548

11. In line 536: the authors say the CRS form is complex. Perhaps they should elaborate more? I do not agree with all the things the CRS form asks (for instance, whether the person smoked or drank alcohol is not collected by most other countries) but I didn't have the impression that it was very complex. If the authors think it is complex, I would like to see an explanation why. Otherwise, I would like this sentence to be reformulated.

Reply- We have removed this sentence in the revised manuscript.

Reviewer #3: I am reviewing this paper from a non-expert perspective - I have limited knowledge of the civil registration system itself, and the specific issues faced in Bihar.

The paper clearly explores an important issue, namely barriers to civil registration in Bihar. The importance of strengthening civil registration has been highlighted by the pandemic, making this study timely. The methodology - interviews with key informants and members of the public - seems appropriate, and the paper includes a number of interesting insights about these barriers at both administrative and cultural levels.

I have a few comments for the authors to consider:

1. Quantitative data. Although this is a qualitative study, it would still be helpful to have some summary quantitative data. At least: amongst the focus group participants in Patna how many births and how many deaths were reported; and how many of these were registered. Plus the same for Vaishali. This is not to draw any quantitative conclusions, but merely to understand the overall sample.

In fact, some quantitative data is presented, but too unclearly. "A few participants (n=15) reported death at the registration centres situated at cemeteries or burial places" - this sentence needs a denominator: 15 out of how many? In sentences such as "Besides, two-fifth of the study participants reported the death of their household members at the block or municipality offices." it would be better to present the actual numbers of how many deaths were reported, and out of these how many at block or municipality offices.

The same comments apply at several other places in the text, for example: "Nearly one-sixth of the total participants were unaware of the death registration procedures and benefits associated with death registration. A higher number of participants from Patna applied for birth and death registration of their household members than participants from Vaishali. Rural and urban differentials in birth and death registration were found." In every case numbers, with denominators, would be better.

"However, a subset of participants (n=10) did not receive a birth certificate for their child at

all." Again, a denominator is needed. Also, not clear: were these people who applied for, but never received, the birth certicificate; or who never applied for the certificate?

Reply- Dear reviewer, we have not explored categorically the number of registered births and deaths, in this study. However, we have shown how many participants registered birth and death of their family members. Besides, we have included denominator in the revised manuscript in light of your suggestions.

2. Context and history of death registration in the state. The authors mention inconsistency in death registration in Bihar - the lack of a clear trend (up to 2019 at least). Do they have any insights as to why there is so much year-to-year variation in the estimated levels of death registration? Are the SRS-based estimates of coverage reliable in their view? What are the factors which could cause a significant drop in registration from one year to the next? If there were changes in the systems in place which affected trends in registration, then this would be interesting to know. Did the interviews provide any hints?

Reply. Our primary study revealed that birth and death registration was decreased during 2013 and 2014 due to majority of CRS staff are engaged in Bihar Assembly election duty. Thereafter, there has been continuous increase in birth and death registration due to introduction of digital registration using CRS portal and awareness campaign.

3. Poverty and marginalisation. I find these sentences problematic: "Lack of birth registration is associated with increased school dropout, child trafficking, and child labor [1,12]. The child mortality rate is higher among unregistered children [13]." It is very likely that the common factor behind these associations is poverty and marginalisation - the sentences are not formally wrong, but suggest the lack of birth registration could be a causal factor behind, say, child trafficking or infant mortality. It seems important to be more clear here.

This raises an important point: the authors really should discuss how factors such as caste and poverty are associated with levels of registration. Are there studies on this topic, relevant to Bihar? If there is a relevant literature, then it should be referenced and discussed at least briefly. It would be interesting to know whether there are examples of other states where poverty and marginalisation are comparable to Bihar, but civil registration is higher and more stable, and if the authors have some insight about why. If many people in Bihar perceive little benefit in reporting deaths, then why is this different in other comparable states? Are the practical barriers - loss of time and income, difficulty, etc., different in other states?

Although the sample may be too small to understand how attitudes to registration vary by caste/class there still seem to be some hints in the data (e.g. not registering infant deaths - "sin of previous birth"). It seems that the authors did not collect demographic data on caste/ occupation/ socioeconomic status, etc? Perhaps the authors should mention this as a limitation of the study. It would still be interesting if they have any insights from this work or the work of others on how these factors might affect access to registration or attitudes to registration.

Reply- We have shown association in the sentence “Lack of birth registration is associated with increased school dropout, child trafficking, and child labor”, not the causal factor. Moreover, we have reframed this sentence in the revised manuscript “A low birth registration is associated with increased school dropout, child trafficking, and child labor”. We did not collect socio-economic data in this study. Also, comparison study with other

similar state cannot be done at present due to limited literature. We have mention this sentence in limitation section of this study. Please see line no. -559-560

4. Recommendations. A number of the recommendations for how to improve vital registration are made by the authors, and many of these could be useful. But it is not really clear where responsibility lies for the failings. After statements like "the officers did not take any steps for improving the registration level in recent times, particularly death registration." could the authors indicate what the officers could have done? Also perhaps there could be more discussion of resources. A "lack of adequate investment" is mentioned in the abstract, and later the key informants "...suggested that adequate funds should be provided for the smooth functioning of the system." What is the resourcing of the CRS in Bihar? Did the interviews provide insight into where such funds should go (more staff? better pay? better training? more registration offices?) Here, again, some comparison with other states would be interesting.

Reply- We have revised the manuscript in light of the suggestions. However, there is limited study on CRS in India, so a comparative study cannot be done at this stage. We will explore this issue in a future study. We have included some suggestions on the role of authority in the recommendation section of this manuscript. Please see line no. 543-551

5. Minor points

"Epidemiological purposes" are mentioned in the introduction - the authors could say more about how, in the context of the current pandemic, there has been an urgent neall-causel cause mortality data on account of weak official surveillance of COVID mortality. Any comments on uncertainties around pandemic mortality in Bihar and in comparable states would help to highlight the current importance of this study.

Reply- We have revised the manuscript in view of suggestions. Please see line no. 452-455

In Table S1, the fertility rates for Bihar and India appear to be exchanged.

Reply- We have cored the Table S1 .Please see supplementary table (S1 table).

Table S1 should perhaps include the estimated death registration level based on SRS and/or NFHS?

Reply- We have included the official estimate of death registration level (Reference-Office of Registrar General of India (ORGI), Report, 2018). ORGI also use SRS death rate for calculating death registration level.

I would suggest the authors carefully reread the manuscript for typographical errors.

Reply- We have revised the manuscript now.
