# Supplementary material for: Performance barriers of Civil Registration System in Bihar: An exploratory study
Source: PLoS One. 2022 Jun 1;17(6):e0268832. doi: 10.1371/journal.pone.0268832 (PMC9159592; doi:10.1371/journal.pone.0268832)
Supplement: Attachment — Submitted filename: Response to reviewers.docx [file pone.0268832.s006.docx]

**Response to reviewers**

Reviewer #1: This is an interesting paper but does not present enough data to support the interpretation or recommendations. The findings support what is already known, and do not provide additional information. It also appears that the authors are not fully aware of the functioning of the CRS system in India. Specific comments follow.

1. Introduction
a. Page 3, paragraph 1 – Health officials and policy makers do not track fertility, mortality and epidemiological patterns using the CRS as suggested by the authors. The Sample Registration System is used for this purpose in India along with the National Family Health Surveys.

Reply- Dear reviewer, We thank you for this observation. However, we wrote the above sentence in global context.

b. Page 4, paragraph 2 – This detailed description of Bihar is not warranted.
c. Page 4, paragraph 3 – This detail is not relevant here. This can be taken to the discussion section while providing the interpretation of the findings.

Reply- We politely disagree with the reviewer. The detailed information of Bihar is required for foreign readers, who are not familier with Bihar. Previous study also included such description in introduction section. (Gopalakrishnan L, Buback L, Fernald L, Walker D, Diamond-Smith N, in addition to The CAS Evaluation Consortium. Using mHealth to improve health care delivery in India: A qualitative examination of the perspectives of community health workers and beneficiaries. PLoS ONE. 2020; 15(1): e0227451). However, we moved adhar card and ration card details to result section (Pg no. 11, line no.-300-306).

2. Methods
a. Figure 1 – In general, it appears that the authors are not fully familiar with the CRS process in the state.
i. Birth and death in a health facility does not directly go to the District office. The process is different for public sector and private sector births and deaths.

Reply- Dear reviewer, during interview with medical staff of private hospital, they revealed that they enter birth or death registration data into CRS portal. However, verification of documents and birth or death certificate are only issued by Nagar Nigam officials. On the other hand, public hospitals can record data into portal and issued certificate directly to individuals. Hospitals data are further transferred to Nagar Nigam, district, and then state level CRS office. We have revised the manuscript now. (see fig 1)

ii. It is also assumed that health facility births/deaths go to Nagar Nigam, which is not the case in villages.

Reply- Dear reviewer, we agree with your observation. In case of villages, villagers report to Aganwadi sevika or block officer directly. However, in urban area, people can report birth or death to Nagar Nigam, block office and hospital when birth or death occurred in hospital. Further, health facilities share the data with Nagar Nigam and district office. PHC in rural areas register birth or death, however, lack of dedicated staff and shortages of registration form limited their capacity to register. We have revised the manuscript now. (Pg no. 10, line no.- 291-294)

iii. Birth and death certificates are issued by the public sector facilities in Bihar.

Reply- Dear reviewer, we agree with your observation. We have revised the manuscript now.

b. Study design and sample
i. A total of 8 blocks were sampled. How were these blocks chosen?

Reply- Dear reviewer, we have explained sampling design in the manuscript. (Pg no.-7, line no.-188-191)

ii. The authors have listed two districts as a small sample for generalization of the study findings. Furthermore, though 8 blocks were selected, the number of staff interviewed is really low (Table 1), which further limits the generalizability of the study.

Reply. Dear reviewer. We believe 15 in-depth interview from randomly selected 8 blocks of two districts could be a representative sample of Bihar population. In additions, we chose Patna and Vaishali districts to investigate how registration practices differ. These districts represent two different urbanization contexts: with Patna being the most urbanized (43%) and Vaishali is the least urbanized district (7%) of Bihar. Moreover, there are total of 105 participants in our study including FGD participants. A previous study was also based on 16 KII and 14 FGD (95 participants). (Oung, M.T., Richter, K., Prasartkul, P. *et al.* Reliable mortality statistics in Myanmar: a qualitative assessment of challenges in two townships. *BMC Public Health* **19,** 356 (2019). <https://doi.org/10.1186/s12889-019-6671-y>.

iii. Page 7, paragraph 1 – The FGD participants were sampled if there was a birth/death in the household in the last 5 years. It is well known that death registration typically is done for males, and in particular when financial transactions are involved. Please specify more details for the sample to interpret the study findings – how many respondents were sampled for birth and how many for death? What was the sex distribution of the births and deceased, and what was the age range of the deceased?

Reply-FGD were conducted to know people’s awareness and barriers to birth and death registration. This study does not aim to present quantitative aspect (demographic characteristics) of birth and death registration.

c. Data collection and analysis
i. When were the data collected? Please specify the time period.

Reply- Dear reviewer, we have mentioned in the manuscript that a primary survey was conducted during February-March 2020 (Pg. no. 5, line no. 133-135).

ii. How many people refused participation?

Reply- About 16 percent (20 people) refused to participate in this study. we have incorporated this sentence in manuscript now. (Pg no. 9, line no. 242-243).

iii. No details are provided about the questions or themes that were explored in the in-depth interviews or the FGDs.

Reply- Dear reviewer, we have attached schedule as additional file 1 to the manuscript now.

.
iv. One author (KK) did data collection, translation from Hindi to English, and then coding. Another author (NS) reviewed these for accuracy. Please provide the details of the review.

Reply- Senior author (NS) reviewed theme, coding and accuracy of the transcript.

3. Results
a. Page 9, last paragraph – It is stated that most participants registered birth through anganwadi sewika and block officers. Does this mean that there were less participants with births in public sector in the FGD, where in the birth can be registered at the facility itself? Most births are delivered in public sector in Bihar.

Reply- During my survey, majority of participant reported that they delivered in public hospital. However, they did not register birth there due to unavailability of dedicated staff at hospital, shortages of registration forms and staff negligence. They reported to Aganwadi worker or block officers when they need birth certificate for seeking child’s school admission or government scheme benefits. (see-pg. no.-10, line no. 291-295)

b. Page 10, paragraph 3 – Law does not mandate registration in 21 days. It is simply that the registration is free within 21 days.

Reply- ORGI report (2021) documented birth and death are to be reported within 21 days of the events. (Office of Registrar General of India (ORGI). Vital Statistics of India based on the Civil Registration System 2019. New Delhi. 2021. <https://censusindia.gov.in/2011-Common/CRS_2019/CRS2019_report.pdf> )

c. Most of the results are presented with “most”, “some”, “fewer”, or “less than half” prefixes and almost no numbers. Qualitative work does not mean that numbers cannot be presented. It should be possible to state the actual numbers from the total sample of respondents.

Reply. We thank you for your observations. We have included participants number in the revised manuscript now. However, a previous qualitative study published in Plosone also showed results without number. (Gopalakrishnan L, Buback L, Fernald L, Walker D, Diamond-Smith N, in addition to The CAS Evaluation Consortium. Using mHealth to improve health care delivery in India: A qualitative examination of the perspectives of community health workers and beneficiaries. PLoS ONE. 2020; 15(1): e0227451)

d. Page 13, last paragraph continued on page 14 – Cause of death is written by a medical doctor, and most deaths do not have a medically certified cause of death. The interpretation of cause of death related study findings is not clear – who is the CRS staff who reported these? Medical doctors? Some other staff? Why will all staff be trained in recording of cause of death?

Reply- We agree with your comments. All staff do not require training in recording of cause of death. Only medical staff associated with CRS may need training on cause of death recording. We have rechecked the transcript and revised the manuscript. (see Pg. no. 15 line no. 431)

e. Page 14 – What is meant by online registration system? Please specify. All block registrar offices are linked to the CRVS portal to provide birth/death certificate.

Reply- Online registration system refers to recording birth or death on real time basis using specific CRS portal connected with internet. All blocks are linked with CRVS portal to provide birth/death certificate however, some blocks lack computer system and dedicated staff for data entry. We have replaced online registration system with CRS portal in revised manuscript.

f. The interpretation of the findings from the CRS staff interviews will have to be done within the CRS context in the state, which falls under the Director of Statistics and Evaluation but the registrars at the local level are under Ministry of Health & Family Welfare or Women Development.

Reply- Dear reviewer, we thank you for the observations. However, registrar at the local level may follow protocols and law as per RBD acts. We understand that most local level registrar (Aganwadi worker or medical staff) do birth and death registration not as primary duty because they are provided with other task by health and welfare departments. However, we have revised the manuscript in light of your comments.

4. Discussion is mostly repetition of the study findings, and the recommendations made are generic which can be made without these data as well.

Reply- This study highlighted cultural barriers to birth or death registration such as name ceremony of child is more important than formal birth registration or death of infant is considered as sin of parents previous birth. Such findings are specific to Bihar culture, which are not widely discussed. This study may provide knowledge to CRS officials to initiate adequate intervention for increasing birth and death registration level. Moreover, the study recommends mandatory burial or cremation permit, which is not followed in lower and middle-income countries and India is not an exception. However, we have revised the discussion section now.

Reviewer #2: This qualitative survey investigates barriers for CRS performance from the perspective of CRS staff and community members from the two purposely districts in Bihar. This paper generally has a merit and will help to design meaningful interventions by considering some of the lessons learned from this in-death information. However, authors need to look into the processes of registration in details. Please see the following points, which can be considered for further modification.

1. It is mentioned that, we purposely selected two districts from Bihar. It is useful to mentioned the reason for this purpose selection. I did not understand why you select Patna and Vishali.

Reply- Dear reviewer, we have purposively selected Patna and Vaishali to present two different context. Patna is highly urnbanised (43 percent) and Vaishali is least urbanised (7 percent). Higher urbanised Patna might have better transport facility, tertiary advanced hospitals and efficient Nagar-Nigam offices as compared to least urbanised Vaishali. We have documented a similar reason for selecting the two districts in the manuscript (Pg no. 7, line no. 180-187).

2. How did you select the sample from community?

Reply- Dear reviewer, all eligible particpants was selected with the help of volunteers (postman, Aganwadi sevika and teacher) and local leaders. (Pg. no. 9, line no.- 235-240).

3. Is there any difference in the process of registration between the place of birth (Public facility delivery, Private facility delivery and Home delivery)? I think, there are some difference in this process. So, when you accessed attitude and perspective authors should consider the thematic area according to these criteria. Similarly, for death as well.

Reply- Dear reviewer, during interview with medical staff of private hospital, they revealed that they enter birth or death registration data into CRS portal. However, verification and birth or death certificate are only issued by Nagar Nigam officials. On the other hand, public hospitals can record data into portal and issue certificate directly to individual. Hospitals data are further transferred to district and then state level CRS office. Further, regarding thematic area, we have revised the manuscript in light of your comments. (see fig1)

4. I think, the introduction should be oriented towards the objective and should be crisp.

Reply- Dear reviewer, we have revised the manuscript in light of your suggestions.

5. CRS 2019 report is now publically available. Consider this report as well.

Reply- We have updated birth and death registration level using CRS 2019 report. (pg. no. 4, line no.- 96-97).

6. Framework figure is more confusing. I suggest for two separate frame work (i.e. Birth registration and death registration separately). This frame work can be made by considering the place of birth and place of death

Reply- We believe birth and death registration could be done from the same CRS offices at all administrative level. Two separate framework may not depict different scenario. However, we have modified the framework. (see fig 1)

7. Key limitations of Civil Registration System (CRS) in India are uniform low reporting of child deaths, low female death registration in many states, and critical gaps in cause of death ascertainment. Did authors collect any information from the staffs that how to reduce this gap and what are their suggestion to increase these death registrations,

Reply- We collected suggestions on reducing gap and increasing registration level from CRS staff. We have incorporated their suggestion in the manuscript (Pg. no.15-16, line.no – 480-492)

8. I would suggest that the discussion should need more re-work specific to the importance of each thematic topic.

Reply- Dear reviewer, we have revised the discussion now.
