# Supplementary material for: Performance barriers of Civil Registration System in Bihar: An exploratory study
Source: PLoS One. 2022 Jun 1;17(6):e0268832. doi: 10.1371/journal.pone.0268832 (PMC9159592; doi:10.1371/journal.pone.0268832)
Supplement: Attachment — Submitted filename: Comments.docx [file pone.0268832.s007.docx]

This is a good piece of scientific work addressing a very important yet neglected issue. Good efforts have been put together to develop this manuscript though it needs some minor revisions.

Major points:

1. Line Nos 121-129: Please make study rationale more clear

2. Figure 1:

a. How are the deaths at 'home' in 'urban' areas registered? Missing in the diagram.

b. How are the deaths in 'hospitals' in rural areas registered? missing in the diagram.

c. Is ASHA/ANM playing any role in death registration as being currently used in Punjab and Haryana? if yes, then mention please. Who are the designated key informants to whom family members can report FIR of an event in both urban and rural areas?

d. At district and state level, which department is responsible for births and deaths registration? Is it Health department like in Haryana and Punjab? These points are not coming out explicitly from the diagram itself!!

3. Please mention three things in each box of Figure 3: Designation of the person under CRS, name of the department the person holds and his/her designation in that department. Example: Like you have mentioned Chief Registrar is the Director of Statistics and Evaluation but of which department???? Please elaborate

4. Did you consider the levels of birth and death registration of district and block as selection criteria for sampling units because that might give a better picture of reasons.

5. The methodology is indicating that the study was conducted among rural aspect of CRS as none of the study participants represented the urban population or municipal corporation. This has undermined the study findings to rural area only. I feel you need to explain this as a study limitation and provide a valid reason for not including the urban representation.

6. In the methodology section, it's important to mention about the levels of birth and death registrations of two districts you have selected. and compare it with other districts.

Minor revisions:

1. Please use one terminology throughout either CRS or CRVS in introduction section

2. Expand ORGI in caption of Figure 1

3. Please correct the spelling of 'anganwadi sevika' throughout the manuscript

4. Mention inthe data collection part of methodology section about the total number of FGDs done

5. Mention inthe data collection part of methodology section about the total number of KII done

6. Line nos 290-93 not clear

7. Please provide supporting data for statement no. 528-529

8. Please provide supporting data for statement no. 338-39

9. Please add references for lines 182-186

10. In line no. 556-57, you have mentioned that birth and death registration is not being done at sub-center or village levle? Is it not community based as of now? Review the lines

11. You have mentioned in line 567 that all PHCs are not linked to CRS. Is this statement true? Please review.

(Director, Alchemist Research and Data Analysis)
