# Supplementary material for: Performance barriers of Civil Registration System in Bihar: An exploratory study
Source: PLoS One. 2022 Jun 1;17(6):e0268832. doi: 10.1371/journal.pone.0268832 (PMC9159592; doi:10.1371/journal.pone.0268832)
Supplement: Attachment — Submitted filename: plosone_crs_bihar.docx [file pone.0268832.s008.docx]

**Review:** Performance barriers of Civil Registration System in Bihar: An exploratory study

This is an interesting exploratory study, relying on interviews with government officials and rural residents, which documents the functioning of the civil registration system in Bihar before the pandemic. Given the importance of understanding and improving the civil registration system, such studies are urgently needed. I felt that the study should have been more in-depth. Interviews with CRS officials and with community members could have been more detailed, and the description of the overall state of the CRS in Bihar, despite the authors’ attempts, is still quite incomplete. However, given the paucity of scholarship on the civil registration system in India, specially of a qualitative nature, this paper is welcome. I have some minor suggestions, which I hope would be helpful for the authors in revising their work.

1. The authors motivate their paper saying that it is important to study the civil registration system in Bihar because of its poor development indicators in general, and the fact that its CRS is estimated to record the lowest proportion of deaths and births. However, although the authors provide some hints at why Bihar’s CRS performs so poorly, we do not get an explicit discussion of why precisely Bihar lacks even other northern Indian states, such as Madhya Pradesh or Rajasthan, which have higher registration coverage than Bihar. In this, the state of Bihar and Uttar Pradesh are way behind all the other states in India, and it is not clear why this is the case. To truly understand this, a comparative design is needed. Perhaps the case is that the lack of linkages between health systems and the CRS which the authors document, or the lack of computerization in Bihar explains why it lags behind other Indian states (even similarly poor ones). My suggestion to the authors is to consider this line of thinking carefully. They can see what the extant literature says about other poor Indian states (they may find looking at the annual CRS reports at the state level and the national level useful here) about CRS being so poor in Bihar. They should also note some lines of thinking for future researchers in the discussion section, as well as perhaps a limitation that they have not adequately addressed these questions.
2. Related to this point, we also don’t get a sense of the changes in the Civil Registration System before the pandemic in Bihar. Existing patterns, both from the data that has been accessed by journalists during the pandemic period (for instance see https://www.indiacovidmapping.org/reports/mortality/BiharFactsheet.pdf), as well as annual estimates before the pandemic suggest improvements in the Civil Registration System in Bihar. How did these improvements came about? If the authors have information on this, it would be extremely valuable. Similarly, what happened to Bihar’s civil registration system during the pandemic? Answering this likely requires fresh data collection. If that is not feasible, then that should be noted in the discussion. If it is possible to call CRS staff on phones and ask some of these questions, then that would be quite valuable. If this is not possible, then perhaps the authors can consider this as a future research endeavor.
3. At a large number of places, I felt that the authors have policy recommendations that were not supported by the information they present. In some cases, their recommendations can actually be harmful. For instance, the authors recommend “mandatory burial or cremation permits.” Instead of improving registration, I can see how this kind of procedure would cause even more problems in a place like Bihar. For instance, people will have to wait to get a death registered and could then only approach burial or cremation grounds? Instead of this, what would work well is a “notification” and “proof” system: burial grounds can notify CRS authorities about a death, and the family could have a slip which would help as a proof of death. These linkages exist in other states, especially with healthcare facilities. Public hospitals can in fact issue death certificates in many states, and private hospitals issue a hospital death certificate (or birth certificate) which can be taken to a registrar to get a death registered.

I was also uncomfortable with the recommendation of fines (lines 533-534). First, the authors already say that there is a fine of Rs. 10 to register a death after 21 days. Worse, the procedure described here to register a death after 21 days looks like a nightmare, with visits to far off offices. More fines when clearly it is the government system that is lacking is a bit counterproductive. The authors should remove this recommendation. In any case, this does not follow from the data presented by the authors.

4. I found the sentence “there was no strict implementation of the birth and death registration law” vague. Which aspects of the law were not strictly implemented, and how was this ascertained?
5. I was not convinced by the literature cited that “lack of birth registration is associated with school dropout …” and so on. It is quite likely that this is just selection, for instance poor children are more likely to drop out and poor children are less likely to have had their births registered. I would suggest dropping this citation.
6. The authors cite the CRS report to report that 7 percent of births and 8 percent of deaths were not registered in 2019. Although this is an official estimate, it is likely an over-estimate. There are multiple reasons for this, among them the fact that the 2019 CRS deaths in this report are compared to 2018 SRS. Other reasons include the fact that the SRS may under-estimate the crude death rate. This line of thinking is explored in detail here (appendix 1: <https://www.medrxiv.org/content/10.1101/2021.09.30.21264376v1>). I leave it to the authors to decide how best to convey the uncertainty in the true rate of registration completion. Perhaps the authors can say “according to official estimates …”.
7. In figure 1, which provides the organization of the CRS, I was surprised that the Panchayat was not mentioned anywhere in the hierarchy. In figure 3, the panchayat sevak is mentioned as a registrar. In some other northern states, such as Madhya Pradesh, the bulk of rural death and birth registration happens at the Panchayat level. Is it the case that this does not happen in Bihar? In Madhya Pradesh, I have seen functioning computers in Panchayat offices which can register deaths. In which case, this may be the reason why death registration is so poor in Bihar – the lack of panchayat registration. The authors could perhaps explore this more, and correct figure 1 if necessary. If the panchayat is not a functional place to register deaths, then this is actually an important policy suggestion, to improve registration and computerization at the Panchayat level.
8. Figure 1 claims that an annual report at the “state level is published by the chief registrar” and also that “Annual report at India and state level is published by ORGI”. This is confusing, and likely the latter sentence needs modification.
9. I thought that the bit about people not reporting infant deaths because it is considered a sin of parents in previous birth was quite revealing. The state government should consider potentially challenging this belief, and this also is a policy recommendation that flows from the findings from the paper. In addition, given the levels of awareness that the authors document, perhaps more information campaigns are also worth recommending.
10. Similarly, I was surprised by the sentence that “Public facilities record all birth and death but don't update on the CTS portal until birth and death certificates are requested by family members.” The authors do not present evidence for it. This is quite important, and also deserves to have highlighting from the perspective of policy recommendation that the paper generates.
11. In line 536: the authors say the CRS form is complex. Perhaps they should elaborate more? I do not agree with all the things the CRS form asks (for instance, whether the person smoked or drank alcohol is not collected by most other countries) but I didn’t have the impression that it was very complex. If the authors think it is complex, I would like to see an explanation why. Otherwise, I would like this sentence to be reformulated.
