# Supplementary material for: Performance barriers of Civil Registration System in Bihar: An exploratory study
Source: PLoS One. 2022 Jun 1;17(6):e0268832. doi: 10.1371/journal.pone.0268832 (PMC9159592; doi:10.1371/journal.pone.0268832)
Supplement: Attachment — Submitted filename: Response to reviewers.docx [file pone.0268832.s011.docx]

**Response to reviewers**

Reviewer #1: Line no. 170-72- Here you are mentioning that 05 persons of given designations were considered for KII. And you had selected four blocks from each district that means 08 blocks in total. so there should have been 40 KIIs instead of 15? Clarify please.

Reply- We thank you for your valuable observation. In this study, i wish to interview Block Statistical Officer (BSO), a Medical Officer, a Panchayat Chief or Ward Parsad (local leader), an Anganwadi Sevika and a registrar at Nagar Nigam as key informants from each block. However, many officials are not available during field study or did not consent for interview. Therefore, we interviewed total 15 key informants based on their availability and their consent for the interview for this study.

Please mention the department and ministry in Figure 3 for all registrars as highlighted in the attached file

Reply- We have revised the fig 3 in light of your suggestions. Please see page no. 7

Line 209: If the questionnaire was semi-structured, then it will have both open and closed ended questions. It cannot be both semi-structured and open ended simultaneously!!

Reply- We have removed the word “semi structured” in the revised manuscript. Please see line no. 209

Line 242-43: Do you think awareness and knowledge of registration procedures is a barrier to well-functioning CRS? Please reframe the sentence correctly.

Reply- We have reframed the sentence in the revised manuscript. Please see line no. 242-243

Line 246-47: Please write the theme which is acting as a critical barrier from these paragraphs.

Reply- We have revised the theme in the revised manuscript. Please see line no. 246-247

Line 250 : How many delivered in public hospital?

Reply- We have included statistics in the revised manuscript. Please see line no. 251.

Line 254: Requiremnt of I-card should be mentioned as "PERCEIVED BARRIER".

Reply- We have revised the manuscript in light of your valuable suggestion. Please see line no. 254-255

Line 297: Did you conducted any analysis for this? Please mention the data

Reply- We did not analyse death registration level by age and sex in this study. Therefore, we have removed this sentence in the revised manuscript.

Line 386-96: I think this is a perceived administrative barrier. May think to shift in theme 2 paragraph

Reply- We have revised the manuscript in light of your valuable suggestion. Please see line no. 396-406.

Line 459-61: Please mention the birth and death registration level of bihar from the latest CRS report

Reply- We have revised the manuscript in light of your valuable suggestion. Please see line no. 464-465

Reviewer #2: Thank you for paying attention to my comments. I still have minor comments, that I believe will strengthen the paper.

- In the objectives (line 33-34), the authors say that "Among all India's states, Bihar showed a lower recording of birth and death in 2019." I think this is not clear, because it can be interpreted as Bihar's death rate being actually lower than other states. Perhaps its better to write: "Bihar had one of the lowest levels of mortality registration among India's states".

Reply- We have revised the manuscript in light of your valuable suggestion. Please see line no. 33-34

- I don't get the point in the results of "death registration was not mandatory for burial or cremation permits." This is as it should be. Waiting for death registration before burial or cremation would be a nightmarish scenario. I really would like this sentence to be removed (I made similar remarks about this appearing elsewhere in the manuscript in the first revision).

Reply- We have removed the sentence in light of your valuable suggestion. Please see line no. 45.

- "a dedicated staff shortage" - you mean shortage of dedicated staff, I am guessing.

Reply- Yes, we mean the same. However, we have reframed the words for clarity. Please see line no. 49

- Fig 1: I am surprised that the aanganawadi sevika can register deaths in rural Bihar. this could actually be a nice system, but its not clear from the paper about the extent to which this functions.

Reply- We have shown work profile of Aganwadi Worker in line no. 408-410. Besides, this study showed Aganwadi workers are one of the most active CRS staff in rural areas.

- Fig 1: similarly, its surprising that for births and deaths in health institutions in Bihar, the health system doesn't generate a birth certificate, and one still has to go to the panchayat secretary. In Fig 3, the authors mention the health system at local area level, but not in fig 1. again, this is quite confusing.

Reply- We have revised fig 1 in the revised manuscript.

- It is revealing that the researchers visited the households with assistance from panchayat secretary. this could lead to biases in reporting, given the power a panchayat secretary has. I feel this should be mentioned as a limitation.

Reply- Panchayat Secretary only guided in finding the eligible households for this study. He had no contribution in FGD and participant’s consent for this study. We assume there was no considerable bias in the study due to panchayat secretary’s assistance.

Reviewer #3: I think the paper is satisfactory and the revisions are adequate.

As a comment for the auhors, I still do feel that the authors could have attempted a summary of the data - e.g., a simple table of how many events (birth or death) occurred amongst the focus group participants, how many were registered (with/without delay), how many of the total births were registered through anganwadi sevikas/block officers, and so forth... this would have been helpful to set the scene. Sentences like "However, two-fifth of the total participants (n=90) applied for their child's birth registration after one year" remain ambiguous unless we know that births occurred in every family.

But perhaps it is not possible for the authors to be more precise if the data gathered does not allow this. And this comment is not critical to the qualitative conclusions of the manuscript.

Reply- I thank you for your valuable suggestions. However, we did not collect detailed quantitative data. It is not possible to show the descriptive statistics of required variable in this study.
